# Der Deutsche Chirurgie Kongress aus Sicht junger Chirurg:innen: Erwartungen und Hürden: Ergebnisse einer bundesweiten Online-Umfrage

**DOI:** 10.1007/s00104-026-02552-8

**Published:** 2026-08-12

**Authors:** Sebastian Schaaf, Stefanie Brunner, Frederik Schlottmann, Sabine Drossard, Benedikt J. Braun, Louisa Schuffert, Dominik Adl Amini, Joscha Mulorz, Romina Rösch, Atrin Pezeshgi, Randa Haraki, Thomas Schmitz-Rixen

**Affiliations:** 1 Klinik für Allgemein‑, Viszeral- und Thoraxchirurgie, Bundeswehrzentralkrankenhaus Koblenz, Rübenacher Str. 170, 56072 Koblenz, Deutschland; 2 Klinik und Poliklinik für Allgemein‑, Viszeral‑, Thorax- und Transplantationschirurgie, Uniklinik Köln, Köln, Deutschland; 3 Klinik für Plastische, Ästhetische, Hand- und Wiederherstellungschirurgie, Medizinische Hochschule Hannover, Hannover, Deutschland; 4 Klinik und Poliklinik für Allgemein‑, Viszeral‑, Transplantations‑, Gefäß- und Kinderchirurgie, Universitätsklinikum Würzburg, Würzburg, Deutschland; 5 Klinik für Unfall- und Wiederherstellungschirurgie, Eberhard-Karls-Universität Tübingen/BG Klinik Tübingen, Tübingen, Deutschland; 6 Klinik und Poliklinik für Kinderchirurgie, Universitätsmedizin Mainz, Mainz, Deutschland; 7 Centrum für Muskuloskeletale Chirurgie, Charité – Universitätsmedizin Berlin, Berlin, Deutschland; 8 Klinik für Gefäß- und Endovaskularchirurgie, Universitätsklinikum Düsseldorf, Düsseldorf, Deutschland; 9 Abteilung für Thoraxchirurgie, Thoraxklinik Heidelberg, Universitätsklinik Heidelberg, Heidelberg, Deutschland; 10 Deutsche Gesellschaft für Chirurgie, Berlin, Deutschland

**Keywords:** Chirurgische Weiterbildung, Nachwuchsförderung, Fort- und Weiterbildung, Mentoring, Kongressformate, Surgical training, Career development, Continuing medical education, Mentoring, Professional conferences

## Abstract

**Hintergrund:**

Der Deutsche Chirurgie Kongress (DCK) stellt eine zentrale Plattform für wissenschaftlichen Austausch und Weiterbildung der chirurgischen Fächer in Deutschland dar. Gleichzeitig wird eine im Verhältnis zur Zielgruppe geringe Teilnahme junger Chirurg:innen beobachtet.

**Ziel der Arbeit (Fragestellung):**

Die Arbeit untersucht Erwartungen, Teilnahmehürden und Verbesserungspotenziale des DCK aus Sicht junger Chirurg:innen.

**Material und Methoden:**

Es wurde eine anonyme, webbasierte Querschnittsumfrage unter jungen Chirurg:innen in Deutschland durchgeführt. Der Fragebogen umfasste soziodemografische Angaben, Kongresserfahrung, Teilnahmehürden, Erwartungen an Inhalte und Formate sowie Fragen zur allgemeinen Wahrnehmung des DCK. Die erfassten quantitativen und qualitativen Daten wurden explorativ ausgewertet.

**Ergebnisse:**

Insgesamt wurden 226 Fragebögen ausgewertet. Eine grundsätzliche Teilnahmebereitschaft bestand bei 90,3 % der Befragten. Als wichtigste Teilnahmehürden wurden fehlende Freistellung (38,1 %), hohe Kosten (22,1 %) sowie unzureichende Information (14,2 %) identifiziert. Inhaltliche Gründe spielten eine untergeordnete Rolle. Besonders hoch priorisiert wurden praxisnahe Formate, wie Hands-on-Kurse (50,9 %), interaktive Sitzungen (33,6 %) sowie Mentoring- und Netzwerkangebote (46,9 %). Die Erwartungen zeigten sich über alle Subgruppen hinweg weitgehend konsistent.

**Diskussion:**

Junge Chirurg:innen berichten von einem hohen Interesse am DCK, ihre Teilnahme wird jedoch durch strukturelle und organisatorische Faktoren limitiert. Die Ergebnisse unterstreichen, dass die Weiterentwicklung des DCK nicht primär in der Erweiterung des Programms, sondern in der gezielten Ausrichtung an den Bedürfnissen junger Chirurg:innen sowie in der Reduktion struktureller Teilnahmehürden liegt. Eine stärkere Ausrichtung auf praxisnahe Weiterbildung, Mentoring, interaktive Formate sowie hybride Konzepte könnte dabei die Attraktivität und Zugänglichkeit des DCK nachhaltig verbessern.

**Graphic abstract:**

**Figure:**
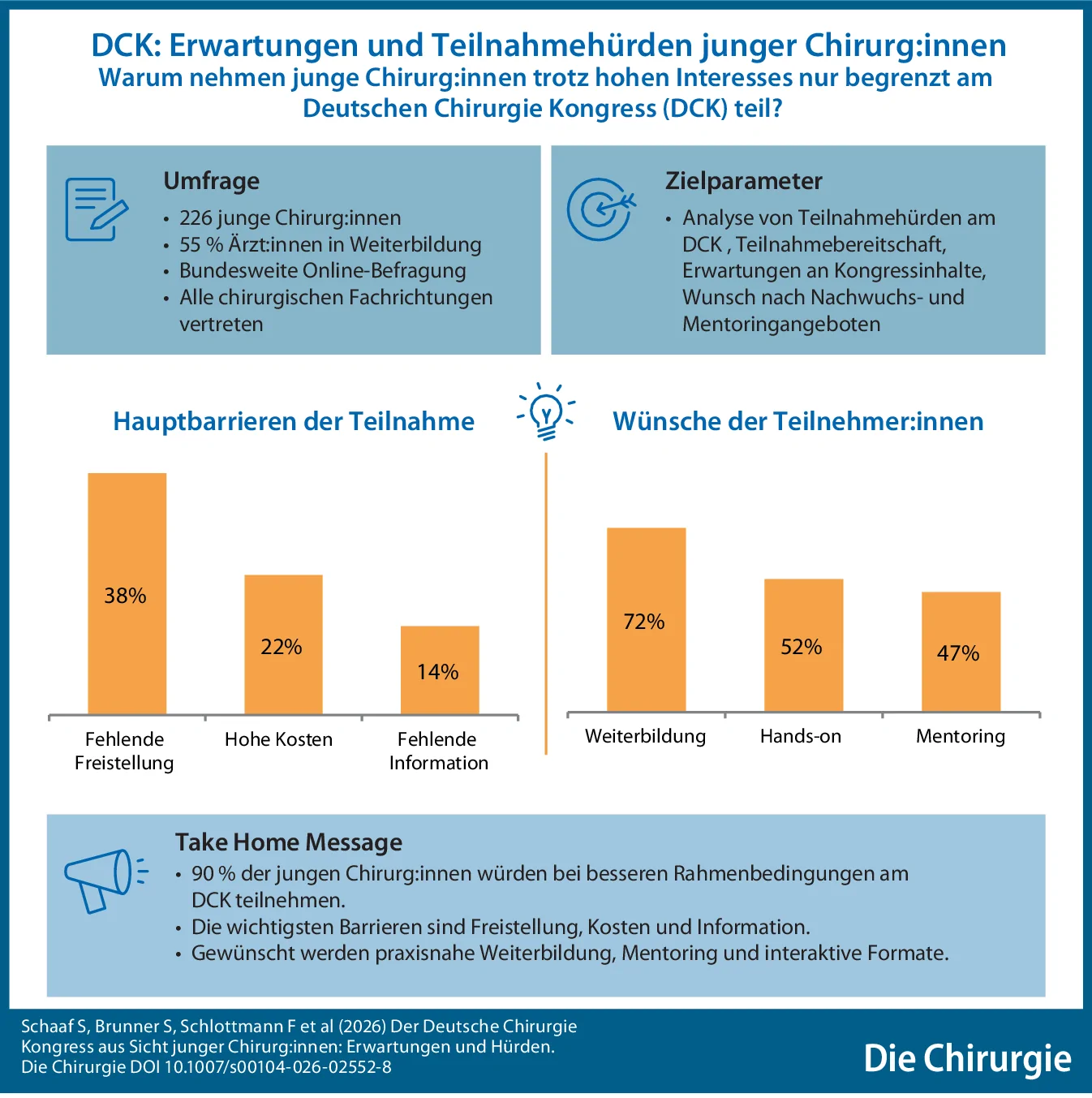

**Zusatzmaterial online:**

Die Online-Version dieses Beitrags (https://doi.org/10.1007/s00104-026-02552-8) enthält die Fragebogenitems mit Antwortmöglichkeiten und die detaillierten Ergebnisse der explorativen Subgruppenanalysen.

## Abstract (en)

**Background:**

The German Surgery Congress (DCK) represents a central platform for scientific exchange and continuing medical education in the surgical disciplines in Germany; however, participation rates among young surgeons appear disproportionately low relative to the target population.

**Objective:**

The study assessed the expectations, barriers to participation and potential areas for improvement of the DCK from the perspective of young surgeons.

**Material and methods:**

An anonymous web-based cross-sectional survey was conducted among young surgeons in Germany. The questionnaire included sociodemographic data, prior congress experience, barriers to participation, expectations regarding content and formats and open-ended responses to general perception of the DCK. The qualitatively and quantitatively collated data were exploratively analyzed.

**Results:**

A total of 226 responses were analyzed. The overall willingness to participate was high (90.3%). The main barriers identified were lack of institutional support/leave (38.1%), high costs (22.1%) and insufficient information about the congress (14.2%). Content-related factors played a minor role. Participants prioritized practice-oriented formats, particularly hands-on courses (50.9%), interactive sessions (33.6%), mentoring and networking opportunities (46.9%). Expectations were largely consistent across subgroups.

**Conclusion:**

Low participation of young surgeons in the DCK appears to be primarily driven by structural and organizational barriers rather than lack of interest. The findings highlight that the further development of the DCK does not primarily depend on expanding the program but on a targeted orientation toward the needs of young surgeons and a reduction In structural access barriers. A stronger emphasis on practice-oriented continuing education, mentoring, interactive formats and hybrid concepts could sustainably improve the attractiveness and accessibility of the DCK.

## Hintergrund und Relevanz

Der Deutsche Chirurgie Kongress (DCK) ist eine zentrale Plattform für wissenschaftlichen Austausch und Weiterbildung in Deutschland. Gleichzeitig steht die chirurgische Weiterbildung vor tiefgreifenden Herausforderungen. Hierzu zählen der demografische Wandel und zunehmende Nachwuchsprobleme, veränderte Erwartungen junger Ärzt:innen an Arbeitszeitmodelle und die Vereinbarkeit von Beruf und Familie, eine steigende Arbeitsverdichtung sowie die Auswirkungen von Krankenhausstrukturreform, Ambulantisierung und ökonomischem Druck auf den klinischen Alltag. Obwohl Kongresse als wichtiger Bestandteil moderner Weiterbildung gelten und die Vernetzung innerhalb des Fachgebiets fördern, ist die Teilnahme junger Chirurg:innen am DCK vergleichsweise gering. Die zugrunde liegenden Ursachen sind bislang nur unzureichend untersucht. Ein besseres Verständnis von Erwartungen, Bedürfnissen und Teilnahmebarrieren ist daher entscheidend, um den DCK gezielt weiterzuentwickeln und seine Attraktivität für den chirurgischen Nachwuchs zu stärken.

## Bedeutung des DCK für Weiterbildung und Nachwuchsförderung

Der Deutsche Chirurgie Kongress (DCK) ist einer der größten chirurgischen Fachkongresse im deutschsprachigen Raum und stellt ein zentrales Forum für wissenschaftlichen Austausch, Fort- und Weiterbildung sowie berufspolitische Diskussion innerhalb der Chirurgie dar. Als Dachkongress der Deutschen Gesellschaft für Chirurgie (DGCH) vereint er die verschiedenen chirurgischen Fachdisziplinen und ermöglicht interdisziplinären Dialog sowie fachübergreifende Vernetzung.

Die chirurgische Weiterbildung in Deutschland steht vor erheblichen strukturellen und demografischen Herausforderungen. Neben einem zunehmenden Fachkräftemangel, veränderten Erwartungen junger Ärzt:innen an Arbeitsbedingungen und Work-Life-Balance sowie einer steigenden Arbeitsverdichtung besteht ein wachsender Bedarf an strukturierter, qualitativ hochwertiger Weiterbildung [2, 11, 12]. Aktuelle Positionspapiere des Perspektivforums Junge Chirurgie (PFJC) und Vertreter:innen der chirurgischen Fachgesellschaften betonen, dass bestehende Defizite in Struktur, Organisation und Finanzierung der Weiterbildung langfristig die Ausbildungsqualität und damit auch die Patientenversorgung/Versorgungsqualität gefährden können [10].

Passend dazu konnte die regelmäßige Teilnahme an Fortbildungen – einschließlich Kongressen – als messbarer Prozessindikator für eine hochwertige chirurgische Weiterbildung identifiziert werden. Kongresse wie der DCK nehmen somit nicht nur eine ergänzende, sondern eine strukturell relevante Rolle innerhalb moderner Weiterbildungskonzepte ein.

Fachkongresse dienen nicht nur der Präsentation wissenschaftlicher Ergebnisse, sondern erfüllen zentrale Funktionen für Weiterbildung, Karriereorientierung, Netzwerkbildung und professionelle Identitätsentwicklung [7]. Gleichzeitig zeigen internationale Entwicklungen, dass klassische Präsenzkongresse zunehmend hinterfragt werden und sich in Richtung praxisnaher, interaktiver und hybrider Formate weiterentwickeln müssen, um insbesondere für jüngere Generationen attraktiv zu bleiben [1]. Auch in Deutschland weist eine aktuelle Befragung von Chirurg:innen darauf hin, dass digitale Technologien eine relevante Rolle für die Attraktivität von Weiterbildungsstrukturen spielen [6].

Parallel dazu weisen aktuelle Analysen darauf hin, dass strukturelle Rahmenbedingungen – insbesondere Arbeitsbelastung, fehlende Freistellung und unzureichende Finanzierung – wesentliche Barrieren für die Teilnahme an Fortbildungsangeboten darstellen [4, 7]. Auch im Kontext der Krankenhausstrukturreform und zunehmenden Ambulantisierung wird die Sicherstellung einer qualitativ hochwertigen Weiterbildung als zentrale Herausforderung beschrieben, die innovative Konzepte und neue Formate erfordert [12]. Die beschriebenen Veränderungen der Versorgungsstrukturen und Arbeitsbedingungen beeinflussen nicht nur die Weiterbildung selbst, sondern potenziell auch die Nutzung etablierter Fortbildungsangebote wie chirurgischer Fachkongresse. Ein besseres Verständnis der Erwartungen und Teilnahmehürden junger Chirurg:innen erscheint daher notwendig, um den DCK als Bestandteil moderner Weiterbildungskonzepte zielgerichtet weiterzuentwickeln.

Trotz der starken Bedeutung des DCK für die Chirurgie in Deutschland liegen bislang keine systematischen Daten zu Erwartungen, Teilnahmehürden und Verbesserungsvorschlägen junger Chirurg:innen in Deutschland vor. Insbesondere fehlt eine differenzierte Analyse, inwieweit strukturelle, organisatorische und inhaltliche Faktoren die Teilnahme beeinflussen.

Vor diesem Hintergrund wurde durch das PFJC der DGCH eine bundesweite, anonyme Online-Umfrage initiiert, um die Perspektive junger Chirurg:innen systematisch zu erfassen. Ziel der vorliegenden Studie ist es, Teilnahmehürden, Erwartungen und Wünsche in Bezug auf den DCK zu analysieren und daraus evidenzbasierte Ansatzpunkte für eine zukunftsgerichtete Weiterentwicklung des Kongresses abzuleiten.

## Material und Methoden

### Studiendesign und Zielgruppe

Es wurde eine anonyme, querschnittliche Online-Umfrage durchgeführt, um Erwartungen, Teilnahmehürden und Verbesserungsvorschläge junger Chirurg:innen in Bezug auf den DCK zu erfassen. Die Befragung richtete sich primär an Ärzt:innen in Weiterbildung sowie an Fachärzt:innen in den ersten Berufsjahren aller chirurgischen Fachrichtungen in Deutschland, einschließlich der Allgemein- und Viszeralchirurgie, Unfallchirurgie und Orthopädie, Kinder- und Jugendchirurgie, Gefäßchirurgie, Thoraxchirurgie, Herzchirurgie, Neurochirurgie, plastischen, rekonstruktiven und ästhetischen Chirurgie sowie der Mund‑, Kiefer- und Gesichtschirurgie.

### Fragebogenentwicklung

Der Fragebogen wurde durch das PFJC der DGCH entwickelt. Die Erstellung erfolgte iterativ auf Basis von Erfahrungen aus der Nachwuchsarbeit sowie informellen Rückmeldungen junger Chirurg:innen. Vor der Anwendung wurde der Fragebogen intern hinsichtlich Verständlichkeit und Plausibilität getestet. Die Ergebnisse dieser Pilotierung wurden nicht in die Analyse einbezogen.

Der vollständige Fragebogen ist im Supplement (Tabelle S1) dargestellt.

Der Fragebogen umfasst 4 Themenbereiche:

1. soziodemografische Angaben (Alter, Geschlecht, beruflicher Status, Fachrichtung, Institution),
2. Kongresserfahrung und Teilnahme am DCK einschließlich Teilnahmehürden und Teilnahmebereitschaft,
3. Erwartungen an Inhalte, Formate und Nachwuchsangebote (fünfstufige Likert-Skala: 1 = unwichtig bis 5 = sehr wichtig) sowie Mehrfachauswahlfragen,
4. allgemeine Bewertung des DCK und offene Freitextantworten.

Mehrfachnennungen waren, sofern gekennzeichnet, möglich. Die durchschnittliche Bearbeitungszeit betrug etwa 10 min.

### Durchführung der Umfrage

Die Datenerhebung erfolgte mittels einer webbasierten Umfrage, die mithilfe von Google Forms durchgeführt wurde. Der Erhebungszeitraum erstreckte sich vom 01.08. bis zum 30.11.2025. Die Rekrutierung der Teilnehmer erfolgte mittels einer E‑Mail-Verteilerliste der DGCH sowie assoziierter Fachgesellschaften, des Netzwerks der Jungen Chirurgie, sozialer Medien und persönlicher Kontakte des PFJC. Die Teilnahme war freiwillig und anonym. Es wurden keine direkt identifizierbaren personenbezogenen Daten erhoben. Zur Minimierung eines Re-Identifikationsrisikos wurden Subgruppen mit weniger als 5 Teilnehmenden nicht gesondert ausgewertet. Unvollständige Datensätze wurden entsprechend der jeweiligen Fragestellung in die Analyse einbezogen; eine technische Kontrolle zur Vermeidung von Mehrfachteilnahmen (z. B. IP-Tracking) erfolgte nicht.

### Datenauswertung und Statistik

Die Auswertung der erhobenen Daten erfolgte mithilfe von Methoden der deskriptiven Statistik und explorativen Datenanalyse. Kategoriale Variablen wurden als absolute und relative Häufigkeiten dargestellt. Für Likert-skalierte Items wurde das Streuungsmaß (Median und Interquartilsabstand [IQR]) angegeben sowie diese Items als kategoriale Variable (Anteil hoher Zustimmung) ausgewertet. Unvollständige Datensätze wurden entsprechend der jeweiligen Fragestellung in die Analyse einbezogen. Für Subgruppenanalysen wurden folgende Gruppen definiert: beruflicher Status (Weiterbildung vs. Fach‑/Oberärzt:innen), Altersgruppen, chirurgische Fachrichtung sowie Institutionstyp. Zur Minimierung eines Re-Identifikationsrisikos wurden Subgruppen mit weniger als 5 Teilnehmenden nicht gesondert ausgewertet. Gruppenvergleiche erfolgten abhängig vom Skalenniveau mittels Mann-Whitney-U-Test (2 Gruppen), Kruskal-Wallis-Test (mehrere Gruppen) sowie Chi-Quadrat-Test für kategoriale Variablen. Ein *p*-Wert < 0,05 wurde als statistisch signifikant gewertet. Aufgrund des explorativen Studiendesigns erfolgte keine Adjustierung für multiples Testen. Die statistische Analyse wurde mit SPSS Version 26.0 (IBM Corp., Armonk, NY, USA) durchgeführt. Freitextantworten wurden mittels qualitativer Inhaltsanalyse thematisch gruppiert. Wiederkehrende Themen wurden identifiziert und durch exemplarische Zitate illustriert.

### Ethik

Die Studie basiert auf einer anonymen, freiwilligen Online-Befragung ohne Erhebung direkt oder indirekt identifizierbarer personenbezogener Daten. Aufgrund der erhobenen Variablen war eine Identifikation einzelner Teilnehmender zu keinem Zeitpunkt möglich, sodass kein Personenbezug im Sinne der Datenschutz-Grundverordnung (DSGVO) vorlag. Vor diesem Hintergrund war gemäß den geltenden nationalen Regularien kein Ethikvotum erforderlich. Die Studie wurde in Übereinstimmung mit den ethischen Grundsätzen der Deklaration von Helsinki durchgeführt. Eine prospektive Studienregistrierung erfolgte nicht, da es sich um eine nichtinterventionelle Befragungsstudie handelt und somit keine Registrierungspflicht gemäß ICMJE-Kriterien besteht. Die Durchführung und Berichterstattung der Online-Befragung orientierten sich an etablierten Empfehlungen für internetbasierte Surveys (CHERRIES).

### Einsatz von künstlicher Intelligenz

Für die sprachliche Überarbeitung des Manuskripts wurden generative KI-Systeme (ChatGPT, OpenAI) eingesetzt, insbesondere zur Unterstützung bei Formulierungen, sprachlicher Glättung, Grammatik- und Stilkorrekturen sowie bei der Strukturierung einzelner Textabschnitte. Die inhaltliche Konzeption der Studie, Datenauswertung, Interpretation der Ergebnisse und die wissenschaftliche Verantwortung für den Beitrag lagen zu jedem Zeitpunkt ausschließlich bei den Autor:innen.

## Ergebnisse

### Soziodemografische Charakteristika

Insgesamt wurden 226 Fragebögen in die Analyse eingeschlossen. Im Jahr 2025 nahmen 5479 Teilnehmer:innen am DCK teil, wovon 517 (9,4 %) als Ärzt:innen in Weiterbildung und 528 (9,6 %) als Student:innen registriert waren (Fa. Wikonect, Wiesbaden). Die Teilnehmenden der vorliegenden Umfrage stellen jedoch keine Stichprobe der DCK-Besucher:innen 2025 dar. Die Befragung richtete sich unabhängig von einer tatsächlichen DCK-Teilnahme an junge Chirurg:innen in Deutschland und schloss sowohl Personen mit als auch ohne bisherige DCK-Erfahrung ein. Die soziodemografischen Charakteristika der Studienteilnehmenden sind in Tab. 1 dargestellt.

**Tab. 1:** Soziodemografische Charakteristika der Studienteilnehmenden (*n* = 226) Darstellung der Verteilung von Alter, Geschlecht, beruflichem Status, chirurgischer Fachrichtung und Institutionstyp. Angaben als absolute Zahlen und Prozentwerte

Merkmal	Kategorie	*n* (%)
Alter	20–24 Jahre	3 (1,3 %)
	25–29 Jahre	35 (15,5 %)
	30–34 Jahre	72 (31,9 %)
	35–40 Jahre	56 (24,8 %)
	> 40 Jahre	60 (26,5 %)
Geschlecht	Weiblich	138 (61,1 %)
	Männlich	84 (37,2 %)
	Divers	1 (0,4 %)
	Keine Angabe	3 (1,3 %)
Berufsstatus	Ärzt:in in Weiterbildung	125 (55,3 %)
	Fachärzt:in	38 (16,8 %)
	Oberärzt:in	37 (16,4 %)
	Sonstige*	< 5 %
Fachrichtung	Allgemein‑/Viszeralchirurgie	164 (72,6 %)
	Kinder- und Jugendchirurgie	20 (8,8 %)
	Unfallchirurgie/Orthopädie	15 (6,6 %)
	Thoraxchirurgie	9 (4,0 %)
	Plastische Chirurgie	7 (3,1 %)
	Gefäßchirurgie	5 (2,2 %)
	Neurochirurgie	3 (1,3 %)
	Mund‑, Kiefer- und Gesichtschirurgie	1 (0,4 %)
	Sonstige (z. B. ACH/TCH, Adipositaschirurgie/Proktologie)	2 (0,8 %)
Institutionstyp	Schwerpunktversorgung	78 (34,5 %)
	Universitätsklinikum	50 (22,1 %)
	Grund- und Regelversorgung	53 (23,5 %)
	Maximalversorgung	39 (17,3 %)
	Sonstige	6 (2,7 %)

### Kongresserfahrung und Teilnahme am DCK

#### Allgemeine Kongresserfahrung

Die Mehrheit der Befragten verfügte bereits über Erfahrung mit medizinisch-wissenschaftlichen Kongressen. Insgesamt gaben 110 Teilnehmende (48,7 %) an, bereits aktiv mit eigenen Beiträgen (z. B. Poster oder Vorträge) teilgenommen zu haben. Weitere 73 (32,3 %) berichteten über eine passive Teilnahme als Zuhörer:innen, während 43 (19,0 %) bislang keine Kongresserfahrung hatten.

In der Subgruppenanalyse zeigte sich ein signifikanter Zusammenhang zwischen beruflichem Status und Kongresserfahrung: Ärzt:innen in Weiterbildung berichteten häufiger über fehlende oder ausschließlich passive Teilnahme, während Fach- und Oberärzt:innen signifikant häufiger aktiv mit eigenen Beiträgen vertreten waren (*p* < 0,001).

In Abhängigkeit von chirurgischer Fachrichtung oder Institutionstyp ergaben sich hingegen keine signifikanten Unterschiede (jeweils *p* > 0,05).

#### Hinderungsgründe für eine Teilnahme am DCK

Unter den 107 Befragten, die bislang nicht am DCK teilgenommen hatten, wurden als häufigste Hinderungsgründe fehlende Freistellung durch die Klinik aufgrund knappen Personals (44/107; 41,1 %), fehlende Kenntnis des Kongresses (31/107; 29,0 %), Freistellung nur unter Einsatz von Urlaub (32/107; 29,9 %) sowie zu hohe Kosten (29/107; 27,1 %) genannt (Tab. 2). Ebenfalls relevant waren fehlende Informationen über den DCK (26/107; 24,3 %).

**Tab. 2:** Gründe für eine bisherige Nichtteilnahme am DCK nach Teilnahmeerfahrung. Vergleich der angegebenen Teilnahmehindernisse zwischen Teilnehmenden mit und ohne vorherige DCK-Teilnahme. Angaben als absolute Zahlen und Prozentwerte; *p*-Werte basieren auf univariaten Gruppenvergleichen

Grund	Nie teilgenommen *n* (%)	Bereits am DCK teilgenommen *n* (%)	*p*-Wert
Keine Freistellung durch die Klinik (knappes Personal)	44 (41,1 %)	42 (35,3 %)	0,411
Keine Freistellung durch die Klinik (Urlaub nehmen notwendig)	32 (29,9 %)	18 (15,1 %)	0,010
Zu hohe Kosten (Teilnahmegebühren, Reise, Unterkunft)	29 (27,1 %)	20 (16,8 %)	0,075
Ich kenne den Kongress nicht	31 (29,0 %)	1 (0,8 %)	< 0,001
Fehlende Informationen über den Kongress	26 (24,3 %)	3 (2,5 %)	< 0,001
Kenne niemanden, der dorthin fährt/wollte nicht alleine fahren	15 (14,0 %)	5 (4,2 %)	0,004
Fehlende Vereinbarkeit mit Familie/privaten Verpflichtungen	13 (12,1 %)	16 (13,4 %)	0,692
Kein konkreter Nutzen für die eigene Weiterbildung	12 (11,2 %)	5 (4,2 %)	0,074
Inhaltliche Themen nicht fachspezifisch genug	10 (9,3 %)	12 (10,1 %)	0,654
Kollision mit parallel stattfindenden Veranstaltungen	10 (9,3 %)	10 (8,4 %)	1,000
Kein Interesse am Format/Inhalt	2 (1,9 %)	4 (3,4 %)	0,686

Im Vergleich zu Befragten, die bereits am DCK teilgenommen hatten, nannten bisherige Nichtteilnehmende signifikant häufiger, den Kongress nicht zu kennen (29,0 % vs. 0,8 %; *p* < 0,001), unzureichend über ihn informiert zu sein (24,3 % vs. 2,5 %; *p* < 0,001) sowie eine notwendige Urlaubnahme aufgrund fehlender Freistellung als Barriere (29,9 % vs. 15,1 %; *p* = 0,010). Auch das Fehlen von Begleitpersonen bzw. sozialer Anbindung wurde in dieser Gruppe häufiger genannt (14,0 % vs. 4,2 %; *p* = 0,004). Dagegen unterschieden sich strukturelle Hürden durch knappes Personal nicht signifikant zwischen den Gruppen (41,1 % vs. 35,3 %; *p* = 0,411).

Diese Ergebnisse sprechen dafür, dass die bisherige Nichtteilnahme am DCK vor allem mit Informationsdefiziten und organisatorischen Zugangshürden assoziiert ist, während allgemeine strukturelle Belastungen wie Personalknappheit auch Teilnehmende mit früherer Kongresserfahrung betreffen.

#### Grundsätzliche Teilnahmebereitschaft

Trotz der genannten Barrieren zeigte sich eine sehr hohe grundsätzliche Bereitschaft zur Teilnahme am DCK. Insgesamt gaben 204 Teilnehmende (90,3 %) an, unter verbesserten Rahmenbedingungen (wieder) teilnehmen zu wollen, während eine grundsätzliche Ablehnung nur selten geäußert wurde (8,0 %).

In der Analyse nach chirurgischer Fachrichtung zeigte sich ein signifikanter Unterschied in der grundsätzlichen Teilnahmebereitschaft (*p* = 0,031). Während die Bereitschaft zur (Wieder‑)Teilnahme in der Allgemein‑/Viszeralchirurgie sowie der Kinder- und Jugendchirurgie sehr hoch war (90–93 %), fiel sie in kleineren Gruppen wie der Unfallchirurgie/Orthopädie oder Thoraxchirurgie numerisch geringer aus. Aufgrund der geringen Fallzahlen einzelner Fachrichtungen sollte dieser Befund jedoch zurückhaltend interpretiert werden. Die Teilnahmebereitschaft war hingegen unabhängig von beruflichem Status, Geschlecht, Alter und Institutionstyp (jeweils *p* > 0,05). Auch in der Subgruppenanalyse nach bisheriger Teilnahmeerfahrung (noch nie, einmalig oder mehrfach) zeigten sich keine signifikanten Unterschiede (*p* > 0,05).

Als wichtigste Voraussetzungen für eine zukünftige Teilnahme wurden insbesondere inhaltliche und strukturelle Verbesserungen genannt. Am häufigsten wurde der Wunsch nach mehr praxisnaher Wissensvermittlung, insbesondere in Form von Kursen und Hands-on-Angeboten, geäußert (50,9 %), gefolgt von interaktiven (33,6 %) und edukativen Formaten (32,3 %).

Digitale Angebote wurden seltener genannt (19,5 % bzw. 16,8 %) und zeigten lediglich nicht signifikante Trends zugunsten jüngerer Teilnehmender und größerer Kliniken (*p* > 0,05).

### Erwartungen an den DCK

#### Bewertung zentraler Kongressaspekte

Die Befragten bewerteten verschiedene für den DCK charakteristische Kongressaspekte, darunter aktuelle wissenschaftliche Themen, praxisnahe Fortbildungskurse, Networking-Möglichkeiten, Karriere- und Mentoringangebote, digitale Formate sowie die aktive Einbindung des chirurgischen Nachwuchses. Insgesamt wurden diese Aspekte überwiegend positiv bewertet. Die Bewertung erfolgte auf einer fünfstufigen Likert-Skala (1 = unwichtig, 5 = sehr wichtig). Die Auswertung zeigt, dass insbesondere aktuelle wissenschaftliche Themen von den Teilnehmenden als wichtig eingeschätzt wurden (Median 5 [Interquartilbereich [IQR] 4–5]; 79,2 % bewerteten diesen Aspekt als eher wichtig bis wichtig [≥ 4]). Auch die Praxisnähe der angebotenen Fortbildungen, der Austausch mit Kolleg:innen sowie klassische Kongressformate wurden überwiegend als wichtig bewertet (jeweils Median 4 [IQR 3–5]; Anteil ≥ 4: ca. 60–74 %). Darüber hinaus wurden der Netzwerkcharakter und der kollegiale Austausch ebenfalls mehrheitlich als wichtig eingeschätzt (Median 4 [IQR 3–5]; ca. 65 % Bewertungen ≥ 4). Gleiches galt für die Aspekte Karriere und Mentoring, die mit einem Median von 4 (IQR 3–5) und einem Anteil von etwa 55 % Bewertungen ≥ 4 als eher wichtig bis wichtig bewertet wurden. Demgegenüber wurden industriebezogene Inhalte sowie teilweise auch digitale Formate seltener als wichtig eingestuft (Median 3 [IQR 2–4]; Anteil ≥ 4: ca. 27–44 %). In den Subgruppenanalysen zeigten sich keine signifikanten Unterschiede in Abhängigkeit von beruflichem Status, Alter, chirurgischer Fachrichtung oder Institutionstyp (jeweils *p* > 0,05).

#### Bevorzugte thematische Schwerpunkte

Als gewünschte thematische Schwerpunkte des DCK wurden insbesondere Inhalte mit direktem Weiterbildungs- und Praxisbezug genannt. Am häufigsten wurden chirurgische Weiterbildungsthemen (*n* = 163; 72,1 %) sowie Hands-on-Workshops und praktische Fertigkeiten (*n* = 117; 51,8 %) ausgewählt, gefolgt von Mentoring- und Netzwerkangeboten (*n* = 106; 46,9 %).

Weitere relevante Themen waren Interdisziplinarität (*n* = 104; 46,0 %), Berufspolitik und Arbeitsbedingungen (*n* = 95; 42,0 %) sowie Digitalisierung und künstliche Intelligenz (*n* = 68; 30,1 %). Auch wissenschaftliche Karrierewege (*n* = 69; 30,5 %), internationale Perspektiven (*n* = 73; 32,3 %) und Gender- und Diversity-Themen (*n* = 61; 27,0 %) wurden von einem relevanten Anteil der Befragten genannt.

Zwischen den Subgruppen ergaben sich keine signifikanten Unterschiede hinsichtlich der gewünschten thematischen Schwerpunkte (jeweils *p* > 0,05).

#### Bevorzugte Veranstaltungsformate

Bezüglich der bevorzugten Formate wurden am häufigsten klassische Vorträge (*n* = 164; 72,6 %) sowie Hands-on-Kurse (*n* = 148; 65,5 %) genannt. Ebenfalls häufig gewünscht waren interaktive Fallbesprechungen (*n* = 129; 57,1 %) und Diskussionsrunden bzw. Panels (*n* = 93; 41,2 %).

Demgegenüber spielten Posterpräsentationen eine deutlich geringere Rolle (*n* = 14; 6,2 %). Damit lagen digitale Formate deutlich hinter klassischen Präsenzformaten wie Vorträgen (72,6 %), Hands-on-Kursen (65,5 %) und interaktiven Fallbesprechungen (57,1 %) zurück.

In den Subgruppenanalysen zeigten sich keine signifikanten Unterschiede in den bevorzugten Formaten in Abhängigkeit von Fachrichtung oder Institutionstyp (jeweils *p* > 0,05). Ärzt:innen in Weiterbildung äußerten tendenziell häufiger Interesse an digitalen Formaten, ohne dass dieser Unterschied statistische Signifikanz erreichte (*p* = 0,37).

#### Bedeutung und Inhalte eines spezifischen Young Surgeons-Programms

Ein spezifisches Angebot für junge Chirurg:innen wurde von der Mehrheit der Befragten als relevant eingeschätzt. Insgesamt bewerteten 121 Teilnehmende (53,5 %) ein solches Programm als sehr wichtig und weitere 56 (24,8 %) als eher wichtig. Demgegenüber stuften 27 (11,9 %) das Angebot als weniger wichtig und 22 (9,7 %) als unwichtig ein. Diese Einschätzung war unabhängig von beruflichem Status, chirurgischer Fachrichtung, Alter und Institutionstyp (jeweils *p* > 0,05).

Als besonders relevante Bestandteile eines „Young-Surgeons“-Programms wurden Workshops zu praktischen Skills (*n* = 173; 76,5 %), Mentoring-Formate mit erfahrenen Chirurg:innen (*n* = 159; 70,4 %) sowie Informationen zu Karrierewegen in der Chirurgie (*n* = 149; 65,9 %) genannt. Weitere häufig genannte Inhalte waren Networking-Veranstaltungen (*n* = 109; 48,2 %), Themen zur Vereinbarkeit von Beruf und Familie (*n* = 105; 46,5 %) sowie berufspolitische Orientierung (*n* = 99; 43,8 %). In den Subgruppenanalysen zeigten sich keine signifikanten Unterschiede in Abhängigkeit von Fachrichtung, Institutionstyp, Alter oder beruflichem Status (jeweils *p* > 0,05).

### Allgemeine Einschätzung des DCK

#### Attraktivität des DCK im Vergleich zu anderen Fachkongressen

Die Attraktivität des DCK im Vergleich zu anderen nationalen oder internationalen chirurgischen Kongressen wurde insgesamt heterogen bewertet; 30 Teilnehmende (13,3 %) stuften den DCK als sehr attraktiv, weitere 38 (16,8 %) als attraktiv ein. Demgegenüber bewerteten 64 Teilnehmende (28,3 %) den DCK als weniger attraktiv und 60 (26,5 %) als unattraktiv; 25 Befragte (11,1 %) äußerten eine neutrale Einschätzung, während ein kleiner Anteil angab, den DCK nicht beurteilen zu können. In der Subgruppenanalyse zeigten sich keine signifikanten Unterschiede in der Attraktivitätsbewertung in Abhängigkeit vom beruflichen Status, vom Alter, von der chirurgischen Fachrichtung oder von der Art des Krankenhauses (jeweils *p* > 0,05).

#### Bewertung der Teilnahmegebühren

Auf die Frage *„Wie bewerten Sie die Teilnahmegebühren des DCK für Ärzt:innen in Weiterbildung (DCK 2025: 120–220€ in Abhängigkeit der Mitgliedschaft in einer Fachgesellschaft)?“* wurden die Gebühren kritisch eingeschätzt. Insgesamt bewerteten 74 Teilnehmende (32,7 %) die Gebühren als zu hoch, während nur 21 (9,3 %) bzw. 29 (12,8 %) diese als sehr gut bzw. angemessen einstuften. Weitere 34 Befragte (15,0 %) bewerteten die Gebühren als ausreichend, und 68 (30,1 %) gaben an, dies nicht beurteilen zu können. In den Subgruppenanalysen zeigten sich keine signifikanten Unterschiede in Abhängigkeit von beruflichem Status, Alter, chirurgischer Fachrichtung oder Institutionstyp (jeweils *p* > 0,05).

#### Bedeutung des Kinderbetreuungsangebots („KidsClub“)

Das angebotene Kinderbetreuungsangebot (KidsClub) wurde nur von einem kleinen Teil der Befragten aktiv genutzt; 104 Teilnehmende (46,0 %) gaben an, keinen Bedarf gehabt zu haben, während 98 (43,3 %) angaben, nicht vom Angebot gewusst zu haben. Unabhängig von der tatsächlichen Nutzung wurde die Bedeutung eines Kinderbetreuungsangebots für die Kongressteilnahme jedoch hoch eingeschätzt; 88 Teilnehmende (38,9 %) bewerteten ein solches Angebot als sehr wichtig, weitere 21 (9,3 %) als eher wichtig; 60 Teilnehmende (26,5 %) stuften es als unwichtig ein. In der Subgruppenanalyse zeigte sich kein signifikanter Zusammenhang zwischen der Bewertung der Kinderbetreuung und Status, Alter, Fachrichtung oder Klinikart (*p* > 0,05).

#### Bewertung von Programmdichte und Parallelveranstaltungen

Die Bewertung der Programmdichte und die Wahrnehmung fachspezifischer Angebote stellen unterschiedliche Aspekte dar. Während 42,0 % der Befragten die hohe Zahl paralleler Sessions kritisierten, bemängelten 7,1 % unabhängig davon ein unzureichendes Angebot an fachspezifischen Inhalten. Beide Kritikpunkte können dabei gleichzeitig bestehen. In der relativen Betrachtung zeigte sich, dass insbesondere Teilnehmende aus kleineren bzw. spezialisierten Fachrichtungen ein Fehlen fachspezifischer Angebote berichteten: Während dieser Aspekt in der Allgemein- und Viszeralchirurgie nur selten genannt wurde (3,7 %), lagen die Anteile in der plastischen Chirurgie (28,6 %), Thoraxchirurgie (22,2 %), Gefäßchirurgie (20,0 %) und Kinderchirurgie (15,0 %) deutlich höher. Die Einschätzung der Balance zwischen allgemeinen und fachspezifischen Kongressinhalten unterschied sich ebenfalls nicht signifikant zwischen den untersuchten Subgruppen hinsichtlich Fachrichtung, Krankenhausart, Alter oder beruflichem Status (jeweils *p* > 0,05).

#### Qualitative Auswertung der Freitextantworten

Insgesamt nutzten 28 Teilnehmende die Möglichkeit zur Angabe freier Verbesserungsvorschläge. Die Antworten ließen sich in mehrere thematische Kategorien clustern (Tab. 3). Im Vordergrund stand insbesondere der Wunsch nach erweiterten digitalen und hybriden Kongressformaten, praxisnahen und wissenschaftlich fundierten Inhalten sowie einem Ausbau von Hands-on- und Weiterbildungsangeboten. Darüber hinaus wurden eine stärkere Einbindung junger Chirurg:innen in die Kongressgestaltung sowie Verbesserungen der Kongresskultur und Diskussionsatmosphäre thematisiert. Auch strukturelle Aspekte wie Organisation, Logistik und die inhaltliche Positionierung des DCK wurden adressiert.

**Tab. 3:** Qualitative Themenbereiche und illustrative Zitate aus Freitextantworten (*n* = 28)

Themenbereich	Zentrale Inhalte	Illustratives Zitat
Digitale & hybride Formate	Wunsch nach Streaming, On-Demand-Zugriff, flexibler Teilnahme	„Auch Aufzeichnungen von Vorträgen/Sessions wären gut, die man on demand ansehen könnte.“
Praxisnähe & wissenschaftliche Qualität	Mehr evidenzbasierte Inhalte, bessere Abstractqualität, strukturierte Weiterbildung	„Mehr wissenschaftliche Vorträge und weniger geladene Vorträge.“
Hands-on & Weiterbildung	Ausbau praktischer Kurse, bessere Zugänglichkeit für Nachwuchs	„Mehr Hands-On Kurse für Studenten/Assis.“
Junge Chirurgie & Mitbestimmung	Stärkere Einbindung in Programm, Moderation und Entscheidungen; weniger Hierarchien	„Mehr Mitsprache durch junge Chirurgie.“
Kongress- & Diskussionskultur	Wunsch nach respektvoller Diskussionsatmosphäre, motivierender Feedbackkultur	„[Situationen], in denen Vortragende respektlos behandelt wurden, wirken abschreckend.“ *(paraphrasiert)*
Mentoring & Networking	Strukturierte Mentoring-Programme, gezielte Networking-Formate	„Austauschformate zwischen jungen und erfahrenen Kolleg:innen wären hilfreich.“ *(paraphrasiert)*
Organisation & Logistik	Programmübersicht, App, Catering, Reisekosten, Stipendien	„Bessere Übersicht über das Programm wäre wünschenswert.“ *(paraphrasiert)*
Struktur & Positionierung des DCK	Fokus auf fachübergreifende Inhalte, klare Abgrenzung zu Fachkongressen	„Der DCK sollte sich auf fachübergreifende Themen konzentrieren.“

## Diskussion

Der DCK ist mit 5479 registrierten Teilnehmer:innen in 2025 einer der größten deutschsprachigen chirurgischen Fachkongresse, wovon knapp 10 % Weiterbildungsassitent:innen waren. Die vorliegende Studie zeigt, dass das grundsätzliche Interesse junger Chirurg:innen an einer Teilnahme am DCK sehr hoch ist, die tatsächliche Teilnahme jedoch maßgeblich durch strukturelle, organisatorische und informationelle Barrieren limitiert wird. Diese Diskrepanz war unabhängig von Fachrichtung, Institutionstyp oder Alter und betraf insbesondere Ärzt:innen in Weiterbildung. Die Ergebnisse sprechen damit gegen mangelndes Interesse als Ursache geringer Teilnahmequoten und vielmehr für systemische Rahmenbedingungen, die eine aktive Kongressteilnahme erschweren.

Die in unserer Studie beobachtete hohe grundsätzliche Teilnahmebereitschaft junger Chirurg:innen steht im Einklang mit früheren Beobachtungen, wonach der Nutzen chirurgischer Fachgesellschaften und ihrer Veranstaltungen grundsätzlich anerkannt wird, die aktive Beteiligung jedoch häufig durch strukturelle Rahmenbedingungen erschwert wird [7, 10, 11]. Die identifizierten Barrieren – insbesondere fehlende Freistellung, Kosten und organisatorische Einschränkungen – decken sich mit internationalen Befunden zur ärztlichen Fort- und Weiterbildung. Mehrere Studien beschreiben Zeitmangel, Arbeitsbelastung und fehlende institutionelle Unterstützung als zentrale, fachrichtungsübergreifende Hemmnisse der Kongressteilnahme [4, 8, 9]. Wichtig erscheint in diesem Kontext auch das prinzipielle Angebot von Kinderbetreuungsangeboten. Neben organisierter Betreuung (KidsClub) könnte bereits der kostenfreie Zugang für eine Betreuungsperson („Nannyprinzip“) auch Eltern sehr kleiner Kinder oder Säuglingen eine Teilnahme ermöglichen, da für diese häufig eine Fremdbetreuung nicht möglich ist.

Als wichtigste Teilnahmehürden wurden auch in unserer Erhebung eine fehlende Freistellung durch die Klinik, hohe Kosten sowie eine unzureichende Information über den Kongress identifiziert. Während finanzielle Aspekte und organisatorische Hürden fachrichtungs- und institutionsübergreifend relevant waren, zeigte sich die mangelnde Kenntnis des DCK signifikant häufiger bei Ärzt:innen in Weiterbildung. Dies weist auf Defizite in der zielgruppenspezifischen Kommunikation hin und legt nahe, dass der DCK für einen Teil der jungen Generation nicht ausreichend sichtbar oder niedrigschwellig zugänglich ist. Der in unserer Studie beobachtete Zusammenhang zwischen mangelnder Kenntnis des DCK und einer geringeren Teilnahme insbesondere bei Ärzt:innen in Weiterbildung unterstreicht die Bedeutung frühzeitiger, zielgruppenspezifischer Information. Vergleichbare Effekte wurden auch außerhalb des chirurgischen Weiterbildungskontexts beschrieben. Hsu et al. konnten zeigen, dass gezielte frühe Berührungspunkte und transparente Information das Interesse und die Wahrnehmung eines chirurgischen Fachgebiets signifikant verbessern können [5].

Die Erwartungen an Inhalte und Formate zeigten sich über alle Subgruppen hinweg bemerkenswert homogen. Praxisnahe Inhalte, Hands-on-Formate, interaktive Sitzungen sowie Mentoring- und Networking-Angebote wurden mit hoher Zustimmung bewertet. Dies spricht für einen breiten Konsens innerhalb der jungen Chirurgie zur Gestaltung moderner Kongressformate. Internationale Studien bestätigen, dass praxisnahe und interaktive Angebote gegenüber klassischen Vortragsformaten bevorzugt werden und insbesondere Mentoringformate einen wichtigen Beitrag zur Karriereorientierung und Nachwuchsförderung leisten [3, 5, 9]. Das Angebot von bereits bestehenden Mentoringinitiativen (z. B. Young ACO [Assoziation Chirurgische Onkologie der DGAV], CAJC [Chirurgische Arbeitsgemeinschaft Junge Chirurgie der DGAV] Mentoring) sollte demnach weiter ausgebaut und promotet werden. Zudem kommt auch dem niedrigschwelligen Networking im Sinne eines gemeinschaftlichen Kongresserlebnisses – insbesondere durch das Gefühl, nicht allein zu sein – eine große Bedeutung für die Identifikation mit dem Fach und der Community zu. Hierzu tragen insbesondere Formate wie Treffen der jungen Foren oder die Young Surgeons Night bei.

Die hohe Priorisierung von Hands-on-Kursen und Skills-Trainings unterstreicht, dass junge Chirurg:innen Kongresse nicht nur als wissenschaftliches Forum, sondern auch als ergänzenden Lernort zur klinischen Ausbildung verstehen. Dies entspricht auch aktuellen Konzepten, die Fortbildungsaktivitäten – einschließlich Kongressteilnahmen – als messbare Qualitätsindikatoren chirurgischer Weiterbildung definieren [11]. Vor dem Hintergrund zunehmender Arbeitsverdichtung und begrenzter Ausbildungszeiten gewinnt diese Funktion weiter an Bedeutung. Der DCK könnte hier eine wichtige Rolle einnehmen, indem er sich stärker als Ort strukturierter, evidenzbasierter Weiterbildung positioniert. Die in unserer Studie beobachtete klare Präferenz für praxisnahe und interaktive Formate wird durch internationale Daten gestützt. Alghamdi et al. zeigten, dass interaktive Kongressformate von Teilnehmenden signifikant höher bewertet werden als traditionelle Vortragsformate, wobei Hands-on-Workshops über alle Erfahrungsstufen hinweg die höchsten Zufriedenheitswerte erzielten [1].

Die Ergebnisse zu digitalen und hybriden Formaten zeigen ein differenzierteres Bild. Zwar wurden digitale Angebote nicht von der Mehrheit als primäre Erwartung genannt, sie wurden jedoch von einem relevanten Anteil der Befragten gewünscht, insbesondere von Ärzt:innen in Weiterbildung. Die qualitativen Freitextantworten verdeutlichen, dass digitale Zugänge v. a. als Möglichkeit zur Teilhabe trotz klinischer Verpflichtungen verstanden werden. Dies spricht für hybride Konzepte, die Präsenzformate ergänzen, ohne deren Mehrwert zu ersetzen. Internationale Kongresse haben in den vergangenen Jahren gezeigt, dass hybride Modelle sowohl Reichweite als auch Nachhaltigkeit erhöhen können. Der in unserer Studie geäußerte Wunsch nach digitalen und hybriden Formaten spiegelt internationale Entwicklungen wider, die zeigen, dass Ärzt:innen insbesondere bei hoher Arbeitsbelastung Online- und hybride Angebote als Möglichkeit sehen, strukturelle Barrieren wie Zeitmangel und geografische Einschränkungen zu überwinden [4, 9]. Interessanterweise zeigen internationale Studien, dass die Zufriedenheit mit Kongressformaten auch vom beruflichen Erfahrungsgrad abhängt. Während jüngere Teilnehmende interaktive Formate besonders positiv bewerten, äußern berufserfahrene Teilnehmende teilweise geringere Zufriedenheit mit modernen Formaten [1]. In der vorliegenden Studie konnten jedoch keine signifikanten Unterschiede in den Präferenzen für Kongressformate in Abhängigkeit von Alter oder beruflichem Status nachgewiesen werden. Dies spricht dafür, dass die in unserer Kohorte geäußerten Erwartungen an praxisnahe, interaktive und teilweise digitale Formate weitgehend fach- und generationsübergreifend geteilt werden.

Ein weiteres zentrales Ergebnis betrifft die allgemeine Einschätzung des DCK. Attraktivität, Programmdichte und Kostenstruktur wurden überwiegend kritisch bewertet – und zwar unabhängig von Fachrichtung, Klinikart oder Status. Insbesondere die hohe Parallelisierung von Sitzungen wurde als hinderlich wahrgenommen. Diese Kritik verweist auf ein Spannungsfeld zwischen inhaltlicher Breite und individueller Zugänglichkeit, was für interdisziplinäre fachgesellschaftsübergreifende Kongresse typisch erscheint. Die Daten legen nahe, dass eine stärkere kuratorische Fokussierung oder modulare Programmstrukturen die wahrgenommene Überforderung reduzieren könnten und damit zur Verbesserung struktureller Qualitätsaspekte von Fortbildungsformaten beitragen könnten [11].

Die qualitative Analyse der Freitextantworten ergänzt die quantitativen Ergebnisse und erlaubt einen tieferen Einblick in die Erwartungen der Teilnehmenden. Neben inhaltlichen und strukturellen Aspekten wurden wiederholt kulturelle Themen angesprochen, darunter die Diskussionskultur, Hierarchien sowie die Sichtbarkeit junger und weiblicher Chirurg:innen. Obwohl diese Aspekte im Rahmen der vorliegenden Studie nicht quantitativ erfasst wurden, deuten die Freitextantworten darauf hin, dass sie für einzelne Teilnehmende bei der Wahrnehmung und Bewertung des Kongresses relevant sein können. Die Ergebnisse legen somit nahe, dass die Weiterentwicklung des DCK neben organisatorischen auch kulturelle Aspekte berücksichtigen sollte. Die in unserer Studie beobachtete hohe grundsätzliche Teilnahmebereitschaft junger Chirurg:innen steht im Einklang mit früheren Beobachtungen, wonach der Nutzen chirurgischer Fachgesellschaften und ihrer Veranstaltungen grundsätzlich anerkannt wird, die aktive Beteiligung jedoch häufig an strukturellen Rahmenbedingungen scheitert [7]. Unsere Ergebnisse unterstreichen, dass junge Chirurg:innen Kongresse nicht ausschließlich als Plattformen zur Wissensvermittlung, sondern auch als wichtige Orte der Karriereorientierung und persönlichen Weiterentwicklung wahrnehmen. In diesem Kontext konnte gezeigt werden, dass Mentoringformate im Rahmen nationaler chirurgischer Kongresse insbesondere Themen wie Karriereplanung, Work-Life-Balance, wissenschaftliche Laufbahnen und berufspolitisches Engagement adressieren und damit zentrale Bedürfnisse junger Chirurg:innen abdecken [3]. Zusammengenommen deuten sowohl unsere Ergebnisse als auch externe Studien darauf hin, dass erfolgreiche Nachwuchsansprache weniger von einzelnen Programmpunkten als von einem kohärenten Gesamtkonzept aus Sichtbarkeit, Praxisnähe, Mentoring und kultureller Offenheit abhängt. Die positiven Effekte früher Expositions- und Mentoringprogramme, wie sie von Hsu et al. beschrieben wurden, lassen sich konzeptionell auch auf große chirurgische Kongresse wie den DCK übertragen [5].

### Limitationen

Die Ergebnisse dieser Studie sind unter Berücksichtigung mehrerer Limitationen zu interpretieren. Aufgrund des freiwilligen Online-Formats ist ein Selektionsbias möglich; insbesondere kongressaffine oder besonders kritische junge Chirurg:innen könnten überproportional vertreten sein. Die Stichprobe ist daher nicht repräsentativ für alle chirurgisch tätigen Ärzt:innen in Deutschland. Zudem waren Ärzt:innen in Weiterbildung sowie Teilnehmende aus der Allgemein- und Viszeralchirurgie überrepräsentiert, wodurch Subgruppenanalysen in kleineren Fachrichtungen nur eingeschränkt belastbar sind.

Die Datenerhebung basiert auf Selbstauskünften, sodass Erinnerungs- und Wahrnehmungsbias nicht ausgeschlossen werden können. Aufgrund des querschnittlichen Studiendesigns sind keine kausalen Schlussfolgerungen möglich. Die statistischen Analysen erfolgten explorativ und ohne Adjustierung für multiples Testen; insbesondere grenzwertige *p*-Werte sollten daher zurückhaltend interpretiert werden.

Trotz dieser Einschränkungen liefert die Studie durch die Kombination quantitativer und qualitativer Analysen differenzierte Einblicke in Erwartungen und Teilnahmehürden junger Chirurg:innen und stellt damit eine belastbare Grundlage für die Weiterentwicklung des DCK dar.

## Schlussfolgerung

Die vorliegende Studie zeigt, dass ein hohes Interesse junger Chirurg:innen an einer Teilnahme am DCK besteht, die tatsächliche Teilnahme jedoch maßgeblich durch strukturelle, organisatorische und informationelle Barrieren limitiert wird. Gleichzeitig besteht ein klarer und konsistenter Wunsch nach praxisnaher, strukturierter Weiterbildung, interaktiven Formaten sowie Mentoring- und Netzwerkangeboten. Die Ergebnisse unterstreichen, dass die Weiterentwicklung des DCK nicht primär in der Erweiterung des Programms, sondern in der gezielten Ausrichtung an den Bedürfnissen junger Chirurg:innen sowie in der Reduktion struktureller Teilnahmehürden liegt.

Aus den Ergebnissen lassen sich in Übereinstimmung mit aktuellen Positionspapieren [10–12] konkrete Empfehlungen für die Weiterentwicklung chirurgischer Kongresse ableiten:

- *Abbau struktureller Teilnahmehürden* (z. B. durch verbesserte Freistellungsregelungen, transparente Kostenstrukturen und institutionelle Unterstützung),
- *stärkere Integration praxisnaher und strukturierter Weiterbildungsformate* (insbesondere Hands-on-Kurse, Skills-Trainings und curricular orientierte Sessions),
- *Förderung interaktiver Formate* (Fallbesprechungen, Diskussionen und kleinere, dialogorientierte Formate anstelle rein passiver Vorträge).
- *systematische Verankerung von Mentoring- und Netzwerkangeboten *(einschließlich strukturierter Programme für junge Chirurg:innen sowie niedrigschwelliger Networking- und Austauschformate zur Förderung der sozialen Integration und Vernetzung),
- *gezielte, frühzeitige und zielgruppenspezifische Kommunikation* (insbesondere zur besseren Erreichbarkeit von Ärzt:innen in Weiterbildung),
- *Ergänzung durch hybride und digitale Formate* (zur Reduktion von Teilnahmebarrieren bei gleichzeitigem Erhalt des Mehrwerts von Präsenzveranstaltungen),
- *Weiterentwicklung der Kongresskultur *(z. B. durch Förderung einer wertschätzenden Diskussions- und Feedbackkultur, stärkere Einbindung junger Chirurg:innen in Programmgestaltung und Moderation sowie Abbau wahrgenommener Hierarchien).

Zusammengenommen deuten sowohl unsere Ergebnisse als auch externe Studien darauf hin, dass erfolgreiche Nachwuchsansprache weniger von einzelnen Programmpunkten als von einem kohärenten Gesamtkonzept aus Sichtbarkeit, Praxisnähe, Mentoring und kultureller Offenheit abhängt.

## Fazit für die Praxis

- Das Interesse junger Chirurg:innen am DCK ist hoch; die tatsächliche Teilnahme wird jedoch insbesondere durch strukturelle, organisatorische und informationelle Hürden erschwert.
- Praxisnahe, interaktive und strukturierte Fortbildungsformate sind zentrale Anforderungen.
- Mentoring- und Networking-Angebote bieten einen wesentlichen Mehrwert.
- Hybride Formate können Teilnahmehürden reduzieren, ohne Präsenzformate zu ersetzen.
- Eine gezielte Ansprache von Ärzt:innen in Weiterbildung ist entscheidend für die Nachwuchseinbindung.

## Supplementary Information

Fragebogenitems mit Antwortmöglichkeiten

Ergebnisse der explorativen Subgruppenanalysen

## Data Availability

Die Datensätze, die im Rahmen der vorliegenden Studie generiert und analysiert wurden, sind auf begründete Anfrage beim korrespondierenden Autor erhältlich.
