# Supplementary material for: The German Surgery Congress from the perspective of young surgeons; expectations and barriers: Results of a nationwide online survey
Source: Chirurgie (Heidelb). 2026 Aug 12;97(10):819–28. [Article in German] doi: 10.1007/s00104-026-02552-8 (PMC13601097; doi:10.1007/s00104-026-02552-8)
Supplement: Supplementary file 1 [file 104_2026_2552_MOESM1_ESM.pdf]

Supplement 1

Tabelle S1: Fragebogenitems mit Antwortmöglichkeiten. Bei Mehrfachauswahlfragen waren mehrere Antworten möglich. Likert-Skalen wurden von 1 (unwichtig) bis 5 (sehr wichtig) kodiert. Freitextantworten wurden qualitativ-inhaltsanalytisch ausgewertet.

Nr.	Themenbereich	Frage	Antwortformat	Antwortoptionen
1	Soziodemografie	Wie alt sind Sie?	Einfachauswahl	20–24 Jahre; 25–29 Jahre; 30–34 Jahre; 35–40 Jahre; >40 Jahre
2	Soziodemografie	Welchem Geschlecht fühlen Sie sich zugehörig?	Einfachauswahl	Weiblich; Männlich; Divers; Keine Angabe
3	Soziodemografie	Welchen beruflichen Status haben Sie derzeit?	Einfachauswahl	Studierende:r; Arzt/Ärztin in Weiterbildung; Facharzt/-ärztin; Oberarzt/-ärztin; Chefarzt/-ärztin; Industrie/Freelance/Sonstiges
4	Soziodemografie	Falls Sie in Weiterbildung sind: In welchem Weiterbildungsjahr befinden Sie sich?	Einfachauswahl	1–2; 3–4; 5–6; >6; Trifft nicht zu
5	Soziodemografie	Welcher chirurgischen Fachrichtung gehören Sie hauptsächlich an?	Einfachauswahl	Allgemein-/Viszeralchirurgie; Unfallchirurgie/Orthopädie; Kinder- und Jugendchirurgie; Plastische Chirurgie; Gefäßchirurgie; Thoraxchirurgie; Herzchirurgie; Neurochirurgie; Mund-, Kiefer- und Gesichtschirurgie; Sonstige
6	Soziodemografie	In welcher Art von Institution arbeiten Sie derzeit?	Einfachauswahl	Universitätsklinikum; Krankenhaus der Maximalversorgung; Krankenhaus der Schwerpunktversorgung

				; Krankenhaus der Grund- und Regelversorgung; Praxis; Industrie; Sonstige
7	Soziodemografie	Träger des Krankenhauses?	Einfachauswahl	Öffentlich; Freigemeinnützig; Privatwirtschaftlich
8	Kongresserfahrung	Haben Sie bereits an Kongressen teilgenommen?	Einfachauswahl	Aktive Teilnahme mit Beitrag; Passive Teilnahme; Nein
9	Kongresserfahrung	Haben Sie bereits am Deutschen Chirurgiekongress (DCK) teilgenommen?	Einfachauswahl	Aktive Teilnahme mit Beitrag; Passive Teilnahme; Nein
10	Teilnahmehürden	Was waren die Hauptgründe dafür, dass Sie bisher nicht am DCK teilgenommen haben?	Mehrfachauswahl	Fehlende Freistellung; Fehlende Kostenübernahme; Zu hohe Kosten; Keine Kenntnis des DCK; Fehlender Nutzen; Fehlendes Interesse; Sonstiges
11	Teilnahmebereitschaft	Würden Sie grundsätzlich gerne (wieder) am DCK teilnehmen, wenn die Rahmenbedingungen besser wären?	Einfachauswahl	Ja; Nein; Unentschlossen
12	Teilnahmebereitschaft	Was müsste sich ändern, damit Sie zukünftig teilnehmen würden?	Mehrfachauswahl	Mehr praxisnahe Kurse; Mehr edukative Sitzungen; Mehr interaktive Sitzungen; Mehr Streaming/Aufzeichnungen; Mehr digitale Begleitformate; Bessere Kinderbetreuung; Verbesserte Vernetzung; Sonstiges
13	Erwartungen	Aktuelle wissenschaftliche Themen	Likert-Skala (1–5)	1 = unwichtig bis 5 = sehr wichtig
14	Erwartungen	Praxisnahe Fortbildungskurse	Likert-Skala (1–5)	1 = unwichtig bis 5 = sehr wichtig
15	Erwartungen	Networking-Möglichkeiten	Likert-Skala (1–5)	1 = unwichtig bis 5 = sehr wichtig

16	Erwartungen	Austausch mit erfahrenen Kolleg:innen	Likert-Skala (1–5)	1 = unwichtig bis 5 = sehr wichtig
17	Erwartungen	Austausch mit jungen Kolleg:innen	Likert-Skala (1–5)	1 = unwichtig bis 5 = sehr wichtig
18	Erwartungen	Karriereförderung / Mentoring-Angebote	Likert-Skala (1–5)	1 = unwichtig bis 5 = sehr wichtig
19	Erwartungen	Industrieausstellung / Produktvorstellungen	Likert-Skala (1–5)	1 = unwichtig bis 5 = sehr wichtig
20	Erwartungen	Digitale Formate (Live-Streams, Mediathek)	Likert-Skala (1–5)	1 = unwichtig bis 5 = sehr wichtig
21	Erwartungen	Video-Sessions zu Standard-Eingriffen	Likert-Skala (1–5)	1 = unwichtig bis 5 = sehr wichtig
22	Erwartungen	Video-Sessions zu komplexen Eingriffen	Likert-Skala (1–5)	1 = unwichtig bis 5 = sehr wichtig
23	Erwartungen	Sessions zum Umgang mit Notfällen	Likert-Skala (1–5)	1 = unwichtig bis 5 = sehr wichtig
24	Erwartungen	Sessions zum Umgang mit Komplikationen	Likert-Skala (1–5)	1 = unwichtig bis 5 = sehr wichtig
25	Erwartungen	Sessions zur Berufspolitik	Likert-Skala (1–5)	1 = unwichtig bis 5 = sehr wichtig
26	Erwartungen	Beteiligung des Nachwuchses durch junge Foren / PFJC	Likert-Skala (1–5)	1 = unwichtig bis 5 = sehr wichtig
27	Erwartungen	Beteiligung des Nachwuchses durch Junior-Vorsitze	Likert-Skala (1–5)	1 = unwichtig bis 5 = sehr wichtig
28	Erwartungen	Beteiligung des Nachwuchses durch Junior-Vorträge	Likert-Skala (1–5)	1 = unwichtig bis 5 = sehr wichtig
29	Erwartungen	Welche Themen sollten künftig stärker auf dem DCK vertreten sein?	Mehrfachauswahl	Chirurgische Weiterbildung & Karrierewege; Hands-on-Workshops; Mentoring & Networking; Interdisziplinarität; Berufspolitik & Arbeitsbedingungen; Digitalisierung & KI; Wissenschaftliche Karrierewege; Gender & Diversity; Internationale Perspektiven;

				Nachhaltigkeit/Klimawandel
30	Erwartungen	Welche Formate bevorzugen Sie für Inhalte auf dem DCK?	Mehrfachauswahl	Vorträge; Hands-on-Kurse; Interaktive Fallbesprechungen; Diskussionsrunden/Panels; Up-to-Date-Sitzungen; Virtuelle Sessions; Posterpräsentationen
31	Young-Surgeons-Programm	Wie wichtig ist Ihnen ein spezielles Angebot für junge Chirurg:innen?	Ordinale Skala	Sehr wichtig; Eher wichtig; Weniger wichtig; Unwichtig
32	Young-Surgeons-Programm	Was sollte ein Young-Surgeons-Programm beinhalten?	Mehrfachauswahl	Praktische Skills-Workshops; Mentoring; Karrierewege; Networking; Vereinbarkeit Familie und Beruf; Berufspolitische Orientierung
33	Allgemeine Einschätzung	Wie attraktiv empfinden Sie den DCK im Vergleich zu anderen Kongressen?	Ordinale Skala	Sehr attraktiv; Attraktiv; Neutral; Weniger attraktiv; Unattraktiv
34	Allgemeine Einschätzung	Wie bewerten Sie die Teilnahmegebühren des DCK für Ärzt:innen in Weiterbildung?	Ordinale Skala	Sehr gut; Gut; Ausreichend; Schlecht; Keine Angabe / nicht beurteilbar
35	Kinderbetreuung	Haben Sie den KidsClub genutzt?	Einfachauswahl	Ja; Nein; Kein Bedarf; Nicht auf dem DCK gewesen
36	Kinderbetreuung	Welche Rolle spielt Kinderbetreuung für Ihre Teilnahmeentscheidung?	Ordinale Skala	Sehr wichtig; Eher wichtig; Teils/teils; Weniger wichtig; Unwichtig
37	Programmbewertung	Wie bewerten Sie die Programmdichte und parallele Sessions?	Einfachauswahl	Zu viele parallele Sessions; Balance passend; Zu wenig Auswahl; Freitextoption
38	Freitext	Weitere Verbesserungsvorschläge oder Wünsche an den DCK	Freitext	Freitextfeld
