# Supplementary material for: The German Surgery Congress from the perspective of young surgeons; expectations and barriers: Results of a nationwide online survey
Source: Chirurgie (Heidelb). 2026 Aug 12;97(10):819–28. [Article in German] doi: 10.1007/s00104-026-02552-8 (PMC13601097; doi:10.1007/s00104-026-02552-8)
Supplement: Supplementary file 2 [file 104_2026_2552_MOESM2_ESM.pdf]

Supplement 2

Tabelle S2 Ergebnisse der explorativen Subgruppenanalysen zu Kongresserfahrung, Teilnahmebereitschaft, Erwartungen an den Deutschen Chirurgie Kongress (DCK), Young-Surgeons-Programm und allgemeinen Einschätzungen. Für kategoriale Variablen wurden Chi-Quadrat-Tests bzw. Fisher-Exact-Tests verwendet, für ordinale Likert-Skalen Mann-Whitney-U-Tests (zwei Gruppen) bzw. Kruskal-Wallis-Tests (mehr als zwei Gruppen). Angegeben sind die Anzahl der analysierten Fälle (n) sowie die jeweiligen p-Werte. Berufsstatus wurde als Assistenzärzt in Weiterbildung (AiW) versus Fach-/Oberärzt dichotomisiert. Die Altersgruppen umfassten ≤29, 30–34, 35–40 und >40 Jahre. Fachrichtungen mit weniger als fünf Teilnehmenden wurden von den entsprechenden Analysen ausgeschlossen. Der Institutionstyp wurde in vier Hauptgruppen zusammengefasst. Aufgrund des explorativen Charakters der Analysen erfolgte keine Adjustierung für multiples Testen; p-Werte <0,05 sind als statistisch signifikant zu interpretieren.

Kategorie	Endpunkt/Frage	Item/Antwortoption	Gruppierung	Test	n analysiert	p-Wert
Kongresserfahrung	Allgemeine Kongresserfahrung		Berufsstatus (AiW vs. Fach-/Oberärzt:innen)	Chi-Quadrat-Test	200	<0,001
Kongresserfahrung	Allgemeine Kongresserfahrung		Fachrichtung (Kategorien ≥5)	Chi-Quadrat-Test	220	0,473
Kongresserfahrung	Allgemeine Kongresserfahrung		Institutionstyp (4 Hauptgruppen)	Chi-Quadrat-Test	220	<0,001
Hinderungsgründe	Hinderungsgründe für eine Teilnahme am DCK	Keine Freistellung durch die Klinik (knappes Personal)	DCK-Teilnahme (nie vs. bereits)	Fisher-Exact-Test	226	0,411
Hinderungsgründe	Hinderungsgründe für eine Teilnahme am DCK	Keine Freistellung durch die Klinik (Urlaub nehmen notwendig)	DCK-Teilnahme (nie vs. bereits)	Fisher-Exact-Test	226	0,010
Hinderungsgründe	Hinderungsgründe für eine Teilnahme am DCK	Zu hohe Kosten (Teilnahmegebühren, Reise, Unterkunft)	DCK-Teilnahme (nie vs. bereits)	Fisher-Exact-Test	226	0,075
Hinderungsgründe	Hinderungsgründe für eine Teilnahme am DCK	Ich kenne den Kongress nicht	DCK-Teilnahme (nie vs. bereits)	Fisher-Exact-Test	226	<0,001

Hinderungsgründe	Hinderungsgründe für eine Teilnahme am DCK	Fehlende Informationen über den Kongress	DCK-Teilnahme (nie vs. bereits)	Fisher-Exact-Test	226	<0,001
Hinderungsgründe	Hinderungsgründe für eine Teilnahme am DCK	Kenne niemanden, der dorthin fährt / wollte nicht alleine fahren	DCK-Teilnahme (nie vs. bereits)	Fisher-Exact-Test	226	0,004
Hinderungsgründe	Hinderungsgründe für eine Teilnahme am DCK	Fehlende Vereinbarkeit mit Familie / privaten Verpflichtungen	DCK-Teilnahme (nie vs. bereits)	Fisher-Exact-Test	226	0,692
Hinderungsgründe	Hinderungsgründe für eine Teilnahme am DCK	Kein konkreter Nutzen für die eigene Weiterbildung	DCK-Teilnahme (nie vs. bereits)	Fisher-Exact-Test	226	0,074
Hinderungsgründe	Hinderungsgründe für eine Teilnahme am DCK	Inhaltliche Themen nicht fachspezifisch genug	DCK-Teilnahme (nie vs. bereits)	Fisher-Exact-Test	226	0,654
Hinderungsgründe	Hinderungsgründe für eine Teilnahme am DCK	Kollision mit parallel stattfindenden Veranstaltungen	DCK-Teilnahme (nie vs. bereits)	Fisher-Exact-Test	226	1,000
Hinderungsgründe	Hinderungsgründe für eine Teilnahme am DCK	Kein Interesse am Format/Inhalt	DCK-Teilnahme (nie vs. bereits)	Fisher-Exact-Test	226	0,686
Hinderungsgründe	Fehlende Freistellung (kombiniert)	knappes Personal oder Urlaub nehmen notwendig	Berufsstatus (AiW vs. Fach-/Oberärzt:innen)	Fisher-Exact-Test	200	0,079
Hinderungsgründe	Fehlende Freistellung (kombiniert)	knappes Personal oder Urlaub nehmen notwendig	Fachrichtung (Kategorien ≥5)	Chi-Quadrat-Test	220	0,051
Hinderungsgründe	Fehlende Freistellung (kombiniert)	knappes Personal oder Urlaub nehmen notwendig	Institutionstyp (4 Hauptgruppen)	Chi-Quadrat-Test	220	0,771
Teilnahmebereitschaft	Grundsätzliche Teilnahmebereitschaft		Berufsstatus (AiW vs. Fach-/Oberärzt:innen)	Chi-Quadrat-Test	200	0,079

Teilnahmebereitschaft	Grundsätzliche Teilnahmebereitschaft		Alter (≤29/30–34/35–40/>40)	Chi-Quadrat-Test	226	0,400
Teilnahmebereitschaft	Grundsätzliche Teilnahmebereitschaft		Geschlecht (weiblich vs. männlich)	Chi-Quadrat-Test	222	0,961
Teilnahmebereitschaft	Grundsätzliche Teilnahmebereitschaft		Fachrichtung (Kategorien ≥5)	Chi-Quadrat-Test	220	0,031
Teilnahmebereitschaft	Grundsätzliche Teilnahmebereitschaft		Institutionstyp (4 Hauptgruppen)	Chi-Quadrat-Test	220	0,288
Teilnahmebereitschaft	Grundsätzliche Teilnahmebereitschaft		DCK-Teilnahme (nie vs. bereits)	Chi-Quadrat-Test	226	0,087
Teilnahmebereitschaft	Voraussetzungen für zukünftige Teilnahme	Mehr wissenschaftliche Sitzungen (u.a. Möglichkeiten zur Präsentation eigener Arbeiten)	Berufsstatus (AiW vs. Fach-/Oberärzt:innen)	Fisher-Exact-Test	200	0,116
Teilnahmebereitschaft	Voraussetzungen für zukünftige Teilnahme	Mehr wissenschaftliche Sitzungen (u.a. Möglichkeiten zur Präsentation eigener Arbeiten)	Alter (≤29/30–34/35–40/>40)	Chi-Quadrat-Test	226	0,191
Teilnahmebereitschaft	Voraussetzungen für zukünftige Teilnahme	Mehr wissenschaftliche Sitzungen (u.a. Möglichkeiten zur Präsentation eigener Arbeiten)	Fachrichtung (Kategorien ≥5)	Chi-Quadrat-Test	220	0,357
Teilnahmebereitschaft	Voraussetzungen für zukünftige Teilnahme	Mehr wissenschaftliche Sitzungen (u.a. Möglichkeiten zur Präsentation eigener Arbeiten)	Institutionstyp (4 Hauptgruppen)	Chi-Quadrat-Test	220	0,316
Teilnahmebereitschaft	Voraussetzungen für zukünftige Teilnahme	Mehr praktische Wissensvermittlung (Kurse, Hands-on-Workshops)	Berufsstatus (AiW vs. Fach-/Oberärzt:innen)	Fisher-Exact-Test	200	<0,001
Teilnahmebereitschaft	Voraussetzungen für zukünftige Teilnahme	Mehr praktische Wissensvermittlung (Kurse, Hands-on-Workshops)	Alter (≤29/30–34/35–40/>40)	Chi-Quadrat-Test	226	<0,001

Teilnahmebereitschaft	Voraussetzungen für zukünftige Teilnahme	Mehr praktische Wissensvermittlung (Kurse, Hands-on-Workshops)	Fachrichtung (Kategorien ≥5)	Chi-Quadrat-Test	220	0,300
Teilnahmebereitschaft	Voraussetzungen für zukünftige Teilnahme	Mehr praktische Wissensvermittlung (Kurse, Hands-on-Workshops)	Institutionstyp (4 Hauptgruppen)	Chi-Quadrat-Test	220	0,552
Teilnahmebereitschaft	Voraussetzungen für zukünftige Teilnahme	Mehr edukative Sitzungen (Propädeutik, Leitlinienvorstellungen)	Berufsstatus (AiW vs. Fach-/Oberärzt:innen)	Fisher-Exact-Test	200	0,434
Teilnahmebereitschaft	Voraussetzungen für zukünftige Teilnahme	Mehr edukative Sitzungen (Propädeutik, Leitlinienvorstellungen)	Alter (≤29/30–34/35–40/>40)	Chi-Quadrat-Test	226	0,997
Teilnahmebereitschaft	Voraussetzungen für zukünftige Teilnahme	Mehr edukative Sitzungen (Propädeutik, Leitlinienvorstellungen)	Fachrichtung (Kategorien ≥5)	Chi-Quadrat-Test	220	0,587
Teilnahmebereitschaft	Voraussetzungen für zukünftige Teilnahme	Mehr edukative Sitzungen (Propädeutik, Leitlinienvorstellungen)	Institutionstyp (4 Hauptgruppen)	Chi-Quadrat-Test	220	0,612
Teilnahmebereitschaft	Voraussetzungen für zukünftige Teilnahme	Mehr interaktive Sitzungen (Falldiskussionen, TED-Abstimmung/Quiz)	Berufsstatus (AiW vs. Fach-/Oberärzt:innen)	Fisher-Exact-Test	200	0,643
Teilnahmebereitschaft	Voraussetzungen für zukünftige Teilnahme	Mehr interaktive Sitzungen (Falldiskussionen, TED-Abstimmung/Quiz)	Alter (≤29/30–34/35–40/>40)	Chi-Quadrat-Test	226	0,147
Teilnahmebereitschaft	Voraussetzungen für zukünftige Teilnahme	Mehr interaktive Sitzungen (Falldiskussionen, TED-Abstimmung/Quiz)	Fachrichtung (Kategorien ≥5)	Chi-Quadrat-Test	220	0,006
Teilnahmebereitschaft	Voraussetzungen für zukünftige Teilnahme	Mehr interaktive Sitzungen (Falldiskussionen, TED-Abstimmung/Quiz)	Institutionstyp (4 Hauptgruppen)	Chi-Quadrat-Test	220	0,794
Teilnahmebereitschaft	Voraussetzungen für zukünftige Teilnahme	Mehr Streaming/Aufzeichnung der Sitzungen des Präsenzkongresses	Berufsstatus (AiW vs. Fach-/Oberärzt:innen)	Fisher-Exact-Test	200	0,590

Teilnahmebereitschaft	Voraussetzungen für zukünftige Teilnahme	Mehr Streaming/Aufzeichnung der Sitzungen des Präsenzkongresses	Alter (≤29/30–34/35–40/>40)	Chi-Quadrat-Test	226	0,602
Teilnahmebereitschaft	Voraussetzungen für zukünftige Teilnahme	Mehr Streaming/Aufzeichnung der Sitzungen des Präsenzkongresses	Fachrichtung (Kategorien ≥5)	Chi-Quadrat-Test	220	0,209
Teilnahmebereitschaft	Voraussetzungen für zukünftige Teilnahme	Mehr Streaming/Aufzeichnung der Sitzungen des Präsenzkongresses	Institutionstyp (4 Hauptgruppen)	Chi-Quadrat-Test	220	0,399
Teilnahmebereitschaft	Voraussetzungen für zukünftige Teilnahme	Ausbau digitaler Vor-Kongress (DCK digital)	Berufsstatus (AiW vs. Fach-/Oberärzt:innen)	Fisher-Exact-Test	200	0,698
Teilnahmebereitschaft	Voraussetzungen für zukünftige Teilnahme	Ausbau digitaler Vor-Kongress (DCK digital)	Alter (≤29/30–34/35–40/>40)	Chi-Quadrat-Test	226	0,309
Teilnahmebereitschaft	Voraussetzungen für zukünftige Teilnahme	Ausbau digitaler Vor-Kongress (DCK digital)	Fachrichtung (Kategorien ≥5)	Chi-Quadrat-Test	220	0,481
Teilnahmebereitschaft	Voraussetzungen für zukünftige Teilnahme	Ausbau digitaler Vor-Kongress (DCK digital)	Institutionstyp (4 Hauptgruppen)	Chi-Quadrat-Test	220	0,005
Teilnahmebereitschaft	Voraussetzungen für zukünftige Teilnahme	Mehr Rahmenprogramm und Networking-Events	Berufsstatus (AiW vs. Fach-/Oberärzt:innen)	Fisher-Exact-Test	200	0,411
Teilnahmebereitschaft	Voraussetzungen für zukünftige Teilnahme	Mehr Rahmenprogramm und Networking-Events	Alter (≤29/30–34/35–40/>40)	Chi-Quadrat-Test	226	0,028
Teilnahmebereitschaft	Voraussetzungen für zukünftige Teilnahme	Mehr Rahmenprogramm und Networking-Events	Fachrichtung (Kategorien ≥5)	Chi-Quadrat-Test	220	0,401
Teilnahmebereitschaft	Voraussetzungen für zukünftige Teilnahme	Mehr Rahmenprogramm und Networking-Events	Institutionstyp (4 Hauptgruppen)	Chi-Quadrat-Test	220	0,735

Teilnahmebereitschaft	Voraussetzungen für zukünftige Teilnahme	(Besseres) Angebot an Kinderbetreuung oder Möglichkeit, Sitzungen hybrid zu verfolgen	Berufsstatus (AiW vs. Fach-/Oberärzt:innen)	Fisher-Exact-Test	200	<0,001
Teilnahmebereitschaft	Voraussetzungen für zukünftige Teilnahme	(Besseres) Angebot an Kinderbetreuung oder Möglichkeit, Sitzungen hybrid zu verfolgen	Alter (≤29/30–34/35–40/>40)	Chi-Quadrat-Test	226	0,021
Teilnahmebereitschaft	Voraussetzungen für zukünftige Teilnahme	(Besseres) Angebot an Kinderbetreuung oder Möglichkeit, Sitzungen hybrid zu verfolgen	Fachrichtung (Kategorien ≥5)	Chi-Quadrat-Test	220	0,711
Teilnahmebereitschaft	Voraussetzungen für zukünftige Teilnahme	(Besseres) Angebot an Kinderbetreuung oder Möglichkeit, Sitzungen hybrid zu verfolgen	Institutionstyp (4 Hauptgruppen)	Chi-Quadrat-Test	220	0,236
Erwartungen	Bewertung zentraler Kongressaspekte (Likert 1–5)	Aktuelle wissenschaftliche Themen	Berufsstatus (AiW vs. Fach-/Oberärzt:innen)	Mann-Whitney-U-Test	200	0,855
Erwartungen	Bewertung zentraler Kongressaspekte (Likert 1–5)	Aktuelle wissenschaftliche Themen	Alter (≤29/30–34/35–40/>40)	Kruskal-Wallis-Test	226	0,427
Erwartungen	Bewertung zentraler Kongressaspekte (Likert 1–5)	Aktuelle wissenschaftliche Themen	Fachrichtung (Kategorien ≥5)	Kruskal-Wallis-Test	220	0,454
Erwartungen	Bewertung zentraler Kongressaspekte (Likert 1–5)	Aktuelle wissenschaftliche Themen	Institutionstyp (4 Hauptgruppen)	Kruskal-Wallis-Test	220	0,390
Erwartungen	Bewertung zentraler Kongressaspekte (Likert 1–5)	Praxisnahe Fortbildungskurse	Berufsstatus (AiW vs. Fach-/Oberärzt:innen)	Mann-Whitney-U-Test	200	<0,001
Erwartungen	Bewertung zentraler Kongressaspekte (Likert 1–5)	Praxisnahe Fortbildungskurse	Alter (≤29/30–34/35–40/>40)	Kruskal-Wallis-Test	226	0,011
Erwartungen	Bewertung zentraler Kongressaspekte (Likert 1–5)	Praxisnahe Fortbildungskurse	Fachrichtung (Kategorien ≥5)	Kruskal-Wallis-Test	220	0,514

Erwartungen	Bewertung zentraler Kongressaspekte (Likert 1–5)	Praxisnahe Fortbildungskurse	Institutionstyp (4 Hauptgruppen)	Kruskal-Wallis-Test	220	0,106
Erwartungen	Bewertung zentraler Kongressaspekte (Likert 1–5)	Networking-Möglichkeiten	Berufsstatus (AiW vs. Fach-/Oberärzt:innen)	Mann-Whitney-U-Test	200	0,649
Erwartungen	Bewertung zentraler Kongressaspekte (Likert 1–5)	Networking-Möglichkeiten	Alter (≤29/30–34/35–40/>40)	Kruskal-Wallis-Test	226	0,151
Erwartungen	Bewertung zentraler Kongressaspekte (Likert 1–5)	Networking-Möglichkeiten	Fachrichtung (Kategorien ≥5)	Kruskal-Wallis-Test	220	0,125
Erwartungen	Bewertung zentraler Kongressaspekte (Likert 1–5)	Networking-Möglichkeiten	Institutionstyp (4 Hauptgruppen)	Kruskal-Wallis-Test	220	0,143
Erwartungen	Bewertung zentraler Kongressaspekte (Likert 1–5)	Austausch mit erfahrenen Kolleg:innen	Berufsstatus (AiW vs. Fach-/Oberärzt:innen)	Mann-Whitney-U-Test	200	0,063
Erwartungen	Bewertung zentraler Kongressaspekte (Likert 1–5)	Austausch mit erfahrenen Kolleg:innen	Alter (≤29/30–34/35–40/>40)	Kruskal-Wallis-Test	226	0,022
Erwartungen	Bewertung zentraler Kongressaspekte (Likert 1–5)	Austausch mit erfahrenen Kolleg:innen	Fachrichtung (Kategorien ≥5)	Kruskal-Wallis-Test	220	0,118
Erwartungen	Bewertung zentraler Kongressaspekte (Likert 1–5)	Austausch mit erfahrenen Kolleg:innen	Institutionstyp (4 Hauptgruppen)	Kruskal-Wallis-Test	220	0,729
Erwartungen	Bewertung zentraler Kongressaspekte (Likert 1–5)	Austausch mit jungen Kolleg:innen	Berufsstatus (AiW vs. Fach-/Oberärzt:innen)	Mann-Whitney-U-Test	200	0,023
Erwartungen	Bewertung zentraler Kongressaspekte (Likert 1–5)	Austausch mit jungen Kolleg:innen	Alter (≤29/30–34/35–40/>40)	Kruskal-Wallis-Test	226	0,023

Erwartungen	Bewertung zentraler Kongressaspekte (Likert 1–5)	Austausch mit jungen Kolleg:innen	Fachrichtung (Kategorien ≥5)	Kruskal-Wallis-Test	220	0,210
Erwartungen	Bewertung zentraler Kongressaspekte (Likert 1–5)	Austausch mit jungen Kolleg:innen	Institutionstyp (4 Hauptgruppen)	Kruskal-Wallis-Test	220	0,463
Erwartungen	Bewertung zentraler Kongressaspekte (Likert 1–5)	Karriereförderung / Mentoring-Angebote	Berufsstatus (AiW vs. Fach-/Oberärzt:innen)	Mann-Whitney-U-Test	200	<0,001
Erwartungen	Bewertung zentraler Kongressaspekte (Likert 1–5)	Karriereförderung / Mentoring-Angebote	Alter (≤29/30–34/35–40/>40)	Kruskal-Wallis-Test	226	<0,001
Erwartungen	Bewertung zentraler Kongressaspekte (Likert 1–5)	Karriereförderung / Mentoring-Angebote	Fachrichtung (Kategorien ≥5)	Kruskal-Wallis-Test	220	0,355
Erwartungen	Bewertung zentraler Kongressaspekte (Likert 1–5)	Karriereförderung / Mentoring-Angebote	Institutionstyp (4 Hauptgruppen)	Kruskal-Wallis-Test	220	0,589
Erwartungen	Bewertung zentraler Kongressaspekte (Likert 1–5)	Industrieausstellung / Produktvorstellungen	Berufsstatus (AiW vs. Fach-/Oberärzt:innen)	Mann-Whitney-U-Test	200	0,484
Erwartungen	Bewertung zentraler Kongressaspekte (Likert 1–5)	Industrieausstellung / Produktvorstellungen	Alter (≤29/30–34/35–40/>40)	Kruskal-Wallis-Test	226	0,006
Erwartungen	Bewertung zentraler Kongressaspekte (Likert 1–5)	Industrieausstellung / Produktvorstellungen	Fachrichtung (Kategorien ≥5)	Kruskal-Wallis-Test	220	0,055
Erwartungen	Bewertung zentraler Kongressaspekte (Likert 1–5)	Industrieausstellung / Produktvorstellungen	Institutionstyp (4 Hauptgruppen)	Kruskal-Wallis-Test	220	0,061
Erwartungen	Bewertung zentraler Kongressaspekte (Likert 1–5)	Digitale Formate (Live-Streams, Mediathek)	Berufsstatus (AiW vs. Fach-/Oberärzt:innen)	Mann-Whitney-U-Test	200	0,816

Erwartungen	Bewertung zentraler Kongressaspekte (Likert 1–5)	Digitale Formate (Live-Streams, Mediathek)	Alter (≤29/30–34/35–40/>40)	Kruskal-Wallis-Test	226	0,022
Erwartungen	Bewertung zentraler Kongressaspekte (Likert 1–5)	Digitale Formate (Live-Streams, Mediathek)	Fachrichtung (Kategorien ≥5)	Kruskal-Wallis-Test	220	0,092
Erwartungen	Bewertung zentraler Kongressaspekte (Likert 1–5)	Digitale Formate (Live-Streams, Mediathek)	Institutionstyp (4 Hauptgruppen)	Kruskal-Wallis-Test	220	0,014
Erwartungen	Bewertung zentraler Kongressaspekte (Likert 1–5)	Video-Sessions zu Standard-Eingriffen (Teaching im Fokus)	Berufsstatus (AiW vs. Fach-/Oberärzt:innen)	Mann-Whitney-U-Test	200	0,053
Erwartungen	Bewertung zentraler Kongressaspekte (Likert 1–5)	Video-Sessions zu Standard-Eingriffen (Teaching im Fokus)	Alter (≤29/30–34/35–40/>40)	Kruskal-Wallis-Test	226	0,363
Erwartungen	Bewertung zentraler Kongressaspekte (Likert 1–5)	Video-Sessions zu Standard-Eingriffen (Teaching im Fokus)	Fachrichtung (Kategorien ≥5)	Kruskal-Wallis-Test	220	0,482
Erwartungen	Bewertung zentraler Kongressaspekte (Likert 1–5)	Video-Sessions zu Standard-Eingriffen (Teaching im Fokus)	Institutionstyp (4 Hauptgruppen)	Kruskal-Wallis-Test	220	0,210
Erwartungen	Bewertung zentraler Kongressaspekte (Likert 1–5)	Video-Sessions zu komplexen Eingriffen (How-I-Do-it im Fokus)	Berufsstatus (AiW vs. Fach-/Oberärzt:innen)	Mann-Whitney-U-Test	200	0,792
Erwartungen	Bewertung zentraler Kongressaspekte (Likert 1–5)	Video-Sessions zu komplexen Eingriffen (How-I-Do-it im Fokus)	Alter (≤29/30–34/35–40/>40)	Kruskal-Wallis-Test	226	0,948
Erwartungen	Bewertung zentraler Kongressaspekte (Likert 1–5)	Video-Sessions zu komplexen Eingriffen (How-I-Do-it im Fokus)	Fachrichtung (Kategorien ≥5)	Kruskal-Wallis-Test	220	0,606
Erwartungen	Bewertung zentraler Kongressaspekte (Likert 1–5)	Video-Sessions zu komplexen Eingriffen (How-I-Do-it im Fokus)	Institutionstyp (4 Hauptgruppen)	Kruskal-Wallis-Test	220	0,045

Erwartungen	Bewertung zentraler Kongressaspekte (Likert 1–5)	Sessions zum Umgang mit Notfällen	Berufsstatus (AiW vs. Fach-/Oberärzt:innen)	Mann-Whitney-U-Test	200	0,005
Erwartungen	Bewertung zentraler Kongressaspekte (Likert 1–5)	Sessions zum Umgang mit Notfällen	Alter (≤29/30–34/35–40/>40)	Kruskal-Wallis-Test	226	0,045
Erwartungen	Bewertung zentraler Kongressaspekte (Likert 1–5)	Sessions zum Umgang mit Notfällen	Fachrichtung (Kategorien ≥5)	Kruskal-Wallis-Test	220	0,291
Erwartungen	Bewertung zentraler Kongressaspekte (Likert 1–5)	Sessions zum Umgang mit Notfällen	Institutionstyp (4 Hauptgruppen)	Kruskal-Wallis-Test	220	<0,001
Erwartungen	Bewertung zentraler Kongressaspekte (Likert 1–5)	Sessions zum Umgang mit Komplikationen	Berufsstatus (AiW vs. Fach-/Oberärzt:innen)	Mann-Whitney-U-Test	200	0,162
Erwartungen	Bewertung zentraler Kongressaspekte (Likert 1–5)	Sessions zum Umgang mit Komplikationen	Alter (≤29/30–34/35–40/>40)	Kruskal-Wallis-Test	226	0,297
Erwartungen	Bewertung zentraler Kongressaspekte (Likert 1–5)	Sessions zum Umgang mit Komplikationen	Fachrichtung (Kategorien ≥5)	Kruskal-Wallis-Test	220	0,193
Erwartungen	Bewertung zentraler Kongressaspekte (Likert 1–5)	Sessions zum Umgang mit Komplikationen	Institutionstyp (4 Hauptgruppen)	Kruskal-Wallis-Test	220	0,008
Erwartungen	Bewertung zentraler Kongressaspekte (Likert 1–5)	Sessions zur Berufspolitik (auch Weiterbildung, Nachwuchsförderung)	Berufsstatus (AiW vs. Fach-/Oberärzt:innen)	Mann-Whitney-U-Test	200	0,443
Erwartungen	Bewertung zentraler Kongressaspekte (Likert 1–5)	Sessions zur Berufspolitik (auch Weiterbildung, Nachwuchsförderung)	Alter (≤29/30–34/35–40/>40)	Kruskal-Wallis-Test	226	0,057
Erwartungen	Bewertung zentraler Kongressaspekte (Likert 1–5)	Sessions zur Berufspolitik (auch Weiterbildung, Nachwuchsförderung)	Fachrichtung (Kategorien ≥5)	Kruskal-Wallis-Test	220	0,470

Erwartungen	Bewertung zentraler Kongressaspekte (Likert 1–5)	Sessions zur Berufspolitik (auch Weiterbildung, Nachwuchsförderung)	Institutionstyp (4 Hauptgruppen)	Kruskal-Wallis-Test	220	0,469
Erwartungen	Bewertung zentraler Kongressaspekte (Likert 1–5)	Aktive Beteiligung des Nachwuchses durch junge Foren der Fachgesellschaften und des Perspektivforums Junge Chirurgie der DGCH	Berufsstatus (AiW vs. Fach-/Oberärzt:innen)	Mann-Whitney-U-Test	200	0,116
Erwartungen	Bewertung zentraler Kongressaspekte (Likert 1–5)	Aktive Beteiligung des Nachwuchses durch junge Foren der Fachgesellschaften und des Perspektivforums Junge Chirurgie der DGCH	Alter (≤29/30–34/35–40/>40)	Kruskal-Wallis-Test	226	0,007
Erwartungen	Bewertung zentraler Kongressaspekte (Likert 1–5)	Aktive Beteiligung des Nachwuchses durch junge Foren der Fachgesellschaften und des Perspektivforums Junge Chirurgie der DGCH	Fachrichtung (Kategorien ≥5)	Kruskal-Wallis-Test	220	0,021
Erwartungen	Bewertung zentraler Kongressaspekte (Likert 1–5)	Aktive Beteiligung des Nachwuchses durch junge Foren der Fachgesellschaften und des Perspektivforums Junge Chirurgie der DGCH	Institutionstyp (4 Hauptgruppen)	Kruskal-Wallis-Test	220	0,384
Erwartungen	Bewertung zentraler Kongressaspekte (Likert 1–5)	Aktive Beteiligung des Nachwuchses durch Junior-Vorsitze	Berufsstatus (AiW vs. Fach-/Oberärzt:innen)	Mann-Whitney-U-Test	200	0,322
Erwartungen	Bewertung zentraler Kongressaspekte (Likert 1–5)	Aktive Beteiligung des Nachwuchses durch Junior-Vorsitze	Alter (≤29/30–34/35–40/>40)	Kruskal-Wallis-Test	226	0,091
Erwartungen	Bewertung zentraler Kongressaspekte (Likert 1–5)	Aktive Beteiligung des Nachwuchses durch Junior-Vorsitze	Fachrichtung (Kategorien ≥5)	Kruskal-Wallis-Test	220	0,372

Erwartungen	Bewertung zentraler Kongressaspekte (Likert 1–5)	Aktive Beteiligung des Nachwuchses durch Junior-Vorsitze	Institutionstyp (4 Hauptgruppen)	Kruskal-Wallis-Test	220	0,369
Erwartungen	Bewertung zentraler Kongressaspekte (Likert 1–5)	Aktive Beteiligung des Nachwuchses durch Junior-Vorträge	Berufsstatus (AiW vs. Fach-/Oberärzt:innen)	Mann-Whitney-U-Test	200	0,373
Erwartungen	Bewertung zentraler Kongressaspekte (Likert 1–5)	Aktive Beteiligung des Nachwuchses durch Junior-Vorträge	Alter (≤29/30–34/35–40/>40)	Kruskal-Wallis-Test	226	0,157
Erwartungen	Bewertung zentraler Kongressaspekte (Likert 1–5)	Aktive Beteiligung des Nachwuchses durch Junior-Vorträge	Fachrichtung (Kategorien ≥5)	Kruskal-Wallis-Test	220	0,496
Erwartungen	Bewertung zentraler Kongressaspekte (Likert 1–5)	Aktive Beteiligung des Nachwuchses durch Junior-Vorträge	Institutionstyp (4 Hauptgruppen)	Kruskal-Wallis-Test	220	0,282
Erwartungen	Bevorzugte thematische Schwerpunkte	Berufspolitik & Arbeitsbedingungen	Berufsstatus (AiW vs. Fach-/Oberärzt:innen)	Fisher-Exact-Test	200	0,238
Erwartungen	Bevorzugte thematische Schwerpunkte	Berufspolitik & Arbeitsbedingungen	Fachrichtung (Kategorien ≥5)	Chi-Quadrat-Test	220	0,903
Erwartungen	Bevorzugte thematische Schwerpunkte	Berufspolitik & Arbeitsbedingungen	Institutionstyp (4 Hauptgruppen)	Chi-Quadrat-Test	220	0,420
Erwartungen	Bevorzugte thematische Schwerpunkte	Chirurgische Weiterbildung & Karrierewege	Berufsstatus (AiW vs. Fach-/Oberärzt:innen)	Fisher-Exact-Test	200	0,003
Erwartungen	Bevorzugte thematische Schwerpunkte	Chirurgische Weiterbildung & Karrierewege	Fachrichtung (Kategorien ≥5)	Chi-Quadrat-Test	220	0,625
Erwartungen	Bevorzugte thematische Schwerpunkte	Chirurgische Weiterbildung & Karrierewege	Institutionstyp (4 Hauptgruppen)	Chi-Quadrat-Test	220	0,244

Erwartungen	Bevorzugte thematische Schwerpunkte	Interdisziplinäre Zusammenarbeit (z. B. Anästhesie, Pflege, Radiologie)	Berufsstatus (AiW vs. Fach-/Oberärzt:innen)	Fisher-Exact-Test	200	1,000
Erwartungen	Bevorzugte thematische Schwerpunkte	Interdisziplinäre Zusammenarbeit (z. B. Anästhesie, Pflege, Radiologie)	Fachrichtung (Kategorien ≥5)	Chi-Quadrat-Test	220	0,240
Erwartungen	Bevorzugte thematische Schwerpunkte	Interdisziplinäre Zusammenarbeit (z. B. Anästhesie, Pflege, Radiologie)	Institutionstyp (4 Hauptgruppen)	Chi-Quadrat-Test	220	0,259
Erwartungen	Bevorzugte thematische Schwerpunkte	Digitalisierung & Künstliche Intelligenz in der Chirurgie	Berufsstatus (AiW vs. Fach-/Oberärzt:innen)	Fisher-Exact-Test	200	0,465
Erwartungen	Bevorzugte thematische Schwerpunkte	Digitalisierung & Künstliche Intelligenz in der Chirurgie	Fachrichtung (Kategorien ≥5)	Chi-Quadrat-Test	220	0,057
Erwartungen	Bevorzugte thematische Schwerpunkte	Digitalisierung & Künstliche Intelligenz in der Chirurgie	Institutionstyp (4 Hauptgruppen)	Chi-Quadrat-Test	220	0,785
Erwartungen	Bevorzugte thematische Schwerpunkte	Ambulantisierung & sektorenübergreifende Versorgung	Berufsstatus (AiW vs. Fach-/Oberärzt:innen)	Fisher-Exact-Test	200	0,078
Erwartungen	Bevorzugte thematische Schwerpunkte	Ambulantisierung & sektorenübergreifende Versorgung	Fachrichtung (Kategorien ≥5)	Chi-Quadrat-Test	220	0,150
Erwartungen	Bevorzugte thematische Schwerpunkte	Ambulantisierung & sektorenübergreifende Versorgung	Institutionstyp (4 Hauptgruppen)	Chi-Quadrat-Test	220	<0,001
Erwartungen	Bevorzugte thematische Schwerpunkte	Qualitätsmanagement & Patientensicherheit	Berufsstatus (AiW vs. Fach-/Oberärzt:innen)	Fisher-Exact-Test	200	0,645
Erwartungen	Bevorzugte thematische Schwerpunkte	Qualitätsmanagement & Patientensicherheit	Fachrichtung (Kategorien ≥5)	Chi-Quadrat-Test	220	0,113

Erwartungen	Bevorzugte thematische Schwerpunkte	Qualitätsmanagement & Patientensicherheit	Institutionstyp (4 Hauptgruppen)	Chi-Quadrat-Test	220	0,840
Erwartungen	Bevorzugte thematische Schwerpunkte	Wissenschaftliche Karriere & Forschungsförderung	Berufsstatus (AiW vs. Fach-/Oberärzt:innen)	Fisher-Exact-Test	200	0,534
Erwartungen	Bevorzugte thematische Schwerpunkte	Wissenschaftliche Karriere & Forschungsförderung	Fachrichtung (Kategorien ≥5)	Chi-Quadrat-Test	220	0,603
Erwartungen	Bevorzugte thematische Schwerpunkte	Wissenschaftliche Karriere & Forschungsförderung	Institutionstyp (4 Hauptgruppen)	Chi-Quadrat-Test	220	<0,001
Erwartungen	Bevorzugte thematische Schwerpunkte	Gender, Diversity & Vereinbarkeit von Familie und Beruf	Berufsstatus (AiW vs. Fach-/Oberärzt:innen)	Fisher-Exact-Test	200	0,631
Erwartungen	Bevorzugte thematische Schwerpunkte	Gender, Diversity & Vereinbarkeit von Familie und Beruf	Fachrichtung (Kategorien ≥5)	Chi-Quadrat-Test	220	0,027
Erwartungen	Bevorzugte thematische Schwerpunkte	Gender, Diversity & Vereinbarkeit von Familie und Beruf	Institutionstyp (4 Hauptgruppen)	Chi-Quadrat-Test	220	0,256
Erwartungen	Bevorzugte thematische Schwerpunkte	Hands-on-Workshops & praxisnahe Kurse	Berufsstatus (AiW vs. Fach-/Oberärzt:innen)	Fisher-Exact-Test	200	0,005
Erwartungen	Bevorzugte thematische Schwerpunkte	Hands-on-Workshops & praxisnahe Kurse	Fachrichtung (Kategorien ≥5)	Chi-Quadrat-Test	220	0,864
Erwartungen	Bevorzugte thematische Schwerpunkte	Hands-on-Workshops & praxisnahe Kurse	Institutionstyp (4 Hauptgruppen)	Chi-Quadrat-Test	220	0,422
Erwartungen	Bevorzugte thematische Schwerpunkte	Nachhaltigkeit und Klimawandel in der Medizin	Berufsstatus (AiW vs. Fach-/Oberärzt:innen)	Fisher-Exact-Test	200	0,866

Erwartungen	Bevorzugte thematische Schwerpunkte	Nachhaltigkeit und Klimawandel in der Medizin	Fachrichtung (Kategorien ≥5)	Chi-Quadrat-Test	220	0,743
Erwartungen	Bevorzugte thematische Schwerpunkte	Nachhaltigkeit und Klimawandel in der Medizin	Institutionstyp (4 Hauptgruppen)	Chi-Quadrat-Test	220	0,284
Erwartungen	Bevorzugte thematische Schwerpunkte	Internationale Perspektiven & globale Chirurgie	Berufsstatus (AiW vs. Fach-/Oberärzt:innen)	Fisher-Exact-Test	200	1,000
Erwartungen	Bevorzugte thematische Schwerpunkte	Internationale Perspektiven & globale Chirurgie	Fachrichtung (Kategorien ≥5)	Chi-Quadrat-Test	220	0,776
Erwartungen	Bevorzugte thematische Schwerpunkte	Internationale Perspektiven & globale Chirurgie	Institutionstyp (4 Hauptgruppen)	Chi-Quadrat-Test	220	0,143
Erwartungen	Bevorzugte thematische Schwerpunkte	Mentoring & Netzwerkmöglichkeiten für junge Chirurg:innen	Berufsstatus (AiW vs. Fach-/Oberärzt:innen)	Fisher-Exact-Test	200	0,081
Erwartungen	Bevorzugte thematische Schwerpunkte	Mentoring & Netzwerkmöglichkeiten für junge Chirurg:innen	Fachrichtung (Kategorien ≥5)	Chi-Quadrat-Test	220	0,799
Erwartungen	Bevorzugte thematische Schwerpunkte	Mentoring & Netzwerkmöglichkeiten für junge Chirurg:innen	Institutionstyp (4 Hauptgruppen)	Chi-Quadrat-Test	220	0,177
Erwartungen	Bevorzugte Veranstaltungsformate	Vorträge	Berufsstatus (AiW vs. Fach-/Oberärzt:innen)	Fisher-Exact-Test	200	0,002
Erwartungen	Bevorzugte Veranstaltungsformate	Vorträge	Fachrichtung (Kategorien ≥5)	Chi-Quadrat-Test	220	0,080
Erwartungen	Bevorzugte Veranstaltungsformate	Vorträge	Institutionstyp (4 Hauptgruppen)	Chi-Quadrat-Test	220	0,156

Erwartungen	Bevorzugte Veranstaltungsformate	Hands-on-Kurse	Berufsstatus (AiW vs. Fach-/Oberärzt:innen)	Fisher-Exact-Test	200	<0,001
Erwartungen	Bevorzugte Veranstaltungsformate	Hands-on-Kurse	Fachrichtung (Kategorien ≥5)	Chi-Quadrat-Test	220	0,371
Erwartungen	Bevorzugte Veranstaltungsformate	Hands-on-Kurse	Institutionstyp (4 Hauptgruppen)	Chi-Quadrat-Test	220	0,974
Erwartungen	Bevorzugte Veranstaltungsformate	Diskussionsrunden/Paneldiskussionen	Berufsstatus (AiW vs. Fach-/Oberärzt:innen)	Fisher-Exact-Test	200	<0,001
Erwartungen	Bevorzugte Veranstaltungsformate	Diskussionsrunden/Paneldiskussionen	Fachrichtung (Kategorien ≥5)	Chi-Quadrat-Test	220	0,432
Erwartungen	Bevorzugte Veranstaltungsformate	Diskussionsrunden/Paneldiskussionen	Institutionstyp (4 Hauptgruppen)	Chi-Quadrat-Test	220	0,191
Erwartungen	Bevorzugte Veranstaltungsformate	Interaktive Fallbesprechungen	Berufsstatus (AiW vs. Fach-/Oberärzt:innen)	Fisher-Exact-Test	200	1,000
Erwartungen	Bevorzugte Veranstaltungsformate	Interaktive Fallbesprechungen	Fachrichtung (Kategorien ≥5)	Chi-Quadrat-Test	220	0,534
Erwartungen	Bevorzugte Veranstaltungsformate	Interaktive Fallbesprechungen	Institutionstyp (4 Hauptgruppen)	Chi-Quadrat-Test	220	0,011
Erwartungen	Bevorzugte Veranstaltungsformate	Posterpräsentationen	Berufsstatus (AiW vs. Fach-/Oberärzt:innen)	Fisher-Exact-Test	200	0,750
Erwartungen	Bevorzugte Veranstaltungsformate	Posterpräsentationen	Fachrichtung (Kategorien ≥5)	Chi-Quadrat-Test	220	0,778

Erwartungen	Bevorzugte Veranstaltungsformate	Posterpräsentationen	Institutionstyp (4 Hauptgruppen)	Chi-Quadrat-Test	220	0,558
Erwartungen	Bevorzugte Veranstaltungsformate	Virtuelle Sessions	Berufsstatus (AiW vs. Fach-/Oberärzt:innen)	Fisher-Exact-Test	200	0,474
Erwartungen	Bevorzugte Veranstaltungsformate	Virtuelle Sessions	Fachrichtung (Kategorien ≥5)	Chi-Quadrat-Test	220	0,837
Erwartungen	Bevorzugte Veranstaltungsformate	Virtuelle Sessions	Institutionstyp (4 Hauptgruppen)	Chi-Quadrat-Test	220	<0,001
Young-Surgeons-Programm	Bedeutung eines spezifischen Young-Surgeons-Programms		Berufsstatus (AiW vs. Fach-/Oberärzt:innen)	Mann-Whitney-U-Test	200	0,006
Young-Surgeons-Programm	Bedeutung eines spezifischen Young-Surgeons-Programms		Alter (≤29/30–34/35–40/>40)	Kruskal-Wallis-Test	226	<0,001
Young-Surgeons-Programm	Bedeutung eines spezifischen Young-Surgeons-Programms		Fachrichtung (Kategorien ≥5)	Kruskal-Wallis-Test	220	0,639
Young-Surgeons-Programm	Bedeutung eines spezifischen Young-Surgeons-Programms		Institutionstyp (4 Hauptgruppen)	Kruskal-Wallis-Test	220	0,176
Young-Surgeons-Programm	Inhalte eines Young-Surgeons-Programms	Workshops zu praktischen Skills (z. B. Nahtkurs, Laparoskopie-Training)	Berufsstatus (AiW vs. Fach-/Oberärzt:innen)	Fisher-Exact-Test	200	0,223
Young-Surgeons-Programm	Inhalte eines Young-Surgeons-Programms	Workshops zu praktischen Skills (z. B. Nahtkurs, Laparoskopie-Training)	Alter (≤29/30–34/35–40/>40)	Chi-Quadrat-Test	226	0,300
Young-Surgeons-Programm	Inhalte eines Young-Surgeons-Programms	Workshops zu praktischen Skills (z. B. Nahtkurs, Laparoskopie-Training)	Fachrichtung (Kategorien ≥5)	Chi-Quadrat-Test	220	0,734

Young-Surgeons-Programm	Inhalte eines Young-Surgeons-Programms	Workshops zu praktischen Skills (z. B. Nahtkurs, Laparoskopie-Training)	Institutionstyp (4 Hauptgruppen)	Chi-Quadrat-Test	220	0,170
Young-Surgeons-Programm	Inhalte eines Young-Surgeons-Programms	Mentoring-Formate mit erfahrenen Chirurg:innen	Berufsstatus (AiW vs. Fach-/Oberärzt:innen)	Fisher-Exact-Test	200	0,424
Young-Surgeons-Programm	Inhalte eines Young-Surgeons-Programms	Mentoring-Formate mit erfahrenen Chirurg:innen	Alter (≤29/30–34/35–40/>40)	Chi-Quadrat-Test	226	0,132
Young-Surgeons-Programm	Inhalte eines Young-Surgeons-Programms	Mentoring-Formate mit erfahrenen Chirurg:innen	Fachrichtung (Kategorien ≥5)	Chi-Quadrat-Test	220	0,467
Young-Surgeons-Programm	Inhalte eines Young-Surgeons-Programms	Mentoring-Formate mit erfahrenen Chirurg:innen	Institutionstyp (4 Hauptgruppen)	Chi-Quadrat-Test	220	0,431
Young-Surgeons-Programm	Inhalte eines Young-Surgeons-Programms	Karrierewege in der Chirurgie: Klinik, Forschung, Ausland, Niederlassung	Berufsstatus (AiW vs. Fach-/Oberärzt:innen)	Fisher-Exact-Test	200	0,643
Young-Surgeons-Programm	Inhalte eines Young-Surgeons-Programms	Karrierewege in der Chirurgie: Klinik, Forschung, Ausland, Niederlassung	Alter (≤29/30–34/35–40/>40)	Chi-Quadrat-Test	226	0,527
Young-Surgeons-Programm	Inhalte eines Young-Surgeons-Programms	Karrierewege in der Chirurgie: Klinik, Forschung, Ausland, Niederlassung	Fachrichtung (Kategorien ≥5)	Chi-Quadrat-Test	220	0,623
Young-Surgeons-Programm	Inhalte eines Young-Surgeons-Programms	Karrierewege in der Chirurgie: Klinik, Forschung, Ausland, Niederlassung	Institutionstyp (4 Hauptgruppen)	Chi-Quadrat-Test	220	0,202
Young-Surgeons-Programm	Inhalte eines Young-Surgeons-Programms	Kurzvorträge „Von Jung für Jung“ – Forschung & Innovation aus dem Nachwuchs	Berufsstatus (AiW vs. Fach-/Oberärzt:innen)	Fisher-Exact-Test	200	0,542
Young-Surgeons-Programm	Inhalte eines Young-Surgeons-Programms	Kurzvorträge „Von Jung für Jung“ – Forschung & Innovation aus dem Nachwuchs	Alter (≤29/30–34/35–40/>40)	Chi-Quadrat-Test	226	0,424

Young-Surgeons-Programm	Inhalte eines Young-Surgeons-Programms	Kurzvorträge „Von Jung für Jung" – Forschung & Innovation aus dem Nachwuchs	Fachrichtung (Kategorien ≥5)	Chi-Quadrat-Test	220	0,595
Young-Surgeons-Programm	Inhalte eines Young-Surgeons-Programms	Kurzvorträge „Von Jung für Jung" – Forschung & Innovation aus dem Nachwuchs	Institutionstyp (4 Hauptgruppen)	Chi-Quadrat-Test	220	0,658
Young-Surgeons-Programm	Inhalte eines Young-Surgeons-Programms	Networking-Veranstaltungen (z. B. Meet-the-Expert, Lunch-Dates, Young Surgeons Night)	Berufsstatus (AiW vs. Fach-/Oberärzt:innen)	Fisher-Exact-Test	200	0,884
Young-Surgeons-Programm	Inhalte eines Young-Surgeons-Programms	Networking-Veranstaltungen (z. B. Meet-the-Expert, Lunch-Dates, Young Surgeons Night)	Alter (≤29/30–34/35–40/>40)	Chi-Quadrat-Test	226	0,156
Young-Surgeons-Programm	Inhalte eines Young-Surgeons-Programms	Networking-Veranstaltungen (z. B. Meet-the-Expert, Lunch-Dates, Young Surgeons Night)	Fachrichtung (Kategorien ≥5)	Chi-Quadrat-Test	220	0,227
Young-Surgeons-Programm	Inhalte eines Young-Surgeons-Programms	Networking-Veranstaltungen (z. B. Meet-the-Expert, Lunch-Dates, Young Surgeons Night)	Institutionstyp (4 Hauptgruppen)	Chi-Quadrat-Test	220	0,355
Young-Surgeons-Programm	Inhalte eines Young-Surgeons-Programms	Berufspolitische Orientierung: Was erwartet uns in der Zukunft?	Berufsstatus (AiW vs. Fach-/Oberärzt:innen)	Fisher-Exact-Test	200	0,882
Young-Surgeons-Programm	Inhalte eines Young-Surgeons-Programms	Berufspolitische Orientierung: Was erwartet uns in der Zukunft?	Alter (≤29/30–34/35–40/>40)	Chi-Quadrat-Test	226	0,642
Young-Surgeons-Programm	Inhalte eines Young-Surgeons-Programms	Berufspolitische Orientierung: Was erwartet uns in der Zukunft?	Fachrichtung (Kategorien ≥5)	Chi-Quadrat-Test	220	0,568
Young-Surgeons-Programm	Inhalte eines Young-Surgeons-Programms	Berufspolitische Orientierung: Was erwartet uns in der Zukunft?	Institutionstyp (4 Hauptgruppen)	Chi-Quadrat-Test	220	0,153
Young-Surgeons-Programm	Inhalte eines Young-Surgeons-Programms	Vereinbarkeit von Beruf und Familie	Berufsstatus (AiW vs. Fach-/Oberärzt:innen)	Fisher-Exact-Test	200	0,468

Young-Surgeons-Programm	Inhalte eines Young-Surgeons-Programms	Vereinbarkeit von Beruf und Familie	Alter (≤29/30–34/35–40/>40)	Chi-Quadrat-Test	226	0,610
Young-Surgeons-Programm	Inhalte eines Young-Surgeons-Programms	Vereinbarkeit von Beruf und Familie	Fachrichtung (Kategorien ≥5)	Chi-Quadrat-Test	220	0,562
Young-Surgeons-Programm	Inhalte eines Young-Surgeons-Programms	Vereinbarkeit von Beruf und Familie	Institutionstyp (4 Hauptgruppen)	Chi-Quadrat-Test	220	0,157
Young-Surgeons-Programm	Inhalte eines Young-Surgeons-Programms	Chirurgie international: Austauschprogramme, globale Gesundheitsprojekte	Berufsstatus (AiW vs. Fach-/Oberärzt:innen)	Fisher-Exact-Test	200	0,145
Young-Surgeons-Programm	Inhalte eines Young-Surgeons-Programms	Chirurgie international: Austauschprogramme, globale Gesundheitsprojekte	Alter (≤29/30–34/35–40/>40)	Chi-Quadrat-Test	226	0,050
Young-Surgeons-Programm	Inhalte eines Young-Surgeons-Programms	Chirurgie international: Austauschprogramme, globale Gesundheitsprojekte	Fachrichtung (Kategorien ≥5)	Chi-Quadrat-Test	220	0,936
Young-Surgeons-Programm	Inhalte eines Young-Surgeons-Programms	Chirurgie international: Austauschprogramme, globale Gesundheitsprojekte	Institutionstyp (4 Hauptgruppen)	Chi-Quadrat-Test	220	0,331
Young-Surgeons-Programm	Inhalte eines Young-Surgeons-Programms	Raum für eigene Anliegen: Open-Mic / offene Diskussionsformate	Berufsstatus (AiW vs. Fach-/Oberärzt:innen)	Fisher-Exact-Test	200	0,001
Young-Surgeons-Programm	Inhalte eines Young-Surgeons-Programms	Raum für eigene Anliegen: Open-Mic / offene Diskussionsformate	Alter (≤29/30–34/35–40/>40)	Chi-Quadrat-Test	226	0,005
Young-Surgeons-Programm	Inhalte eines Young-Surgeons-Programms	Raum für eigene Anliegen: Open-Mic / offene Diskussionsformate	Fachrichtung (Kategorien ≥5)	Chi-Quadrat-Test	220	0,831
Young-Surgeons-Programm	Inhalte eines Young-Surgeons-Programms	Raum für eigene Anliegen: Open-Mic / offene Diskussionsformate	Institutionstyp (4 Hauptgruppen)	Chi-Quadrat-Test	220	0,011

Allgemeine Einschätzung	Attraktivität des DCK im Vergleich zu anderen Fachkongressen	Berufsstatus (AiW vs. Fach-/Oberärzt:innen)	Mann-Whitney-U-Test	163	0,138
Allgemeine Einschätzung	Attraktivität des DCK im Vergleich zu anderen Fachkongressen	Alter (≤29/30–34/35–40/>40)	Kruskal-Wallis-Test	188	0,603
Allgemeine Einschätzung	Attraktivität des DCK im Vergleich zu anderen Fachkongressen	Fachrichtung (Kategorien ≥5)	Kruskal-Wallis-Test	183	0,142
Allgemeine Einschätzung	Attraktivität des DCK im Vergleich zu anderen Fachkongressen	Institutionstyp (4 Hauptgruppen)	Kruskal-Wallis-Test	183	0,557
Allgemeine Einschätzung	Bewertung der Teilnahmegebühren	Berufsstatus (AiW vs. Fach-/Oberärzt:innen)	Mann-Whitney-U-Test	174	0,183
Allgemeine Einschätzung	Bewertung der Teilnahmegebühren	Alter (≤29/30–34/35–40/>40)	Kruskal-Wallis-Test	197	0,351
Allgemeine Einschätzung	Bewertung der Teilnahmegebühren	Fachrichtung (Kategorien ≥5)	Kruskal-Wallis-Test	192	0,506
Allgemeine Einschätzung	Bewertung der Teilnahmegebühren	Institutionstyp (4 Hauptgruppen)	Kruskal-Wallis-Test	191	0,253
Allgemeine Einschätzung	Bedeutung des Kinderbetreuungsangebots (KidsClub)	Berufsstatus (AiW vs. Fach-/Oberärzt:innen)	Mann-Whitney-U-Test	200	0,831
Allgemeine Einschätzung	Bedeutung des Kinderbetreuungsangebots (KidsClub)	Alter (≤29/30–34/35–40/>40)	Kruskal-Wallis-Test	226	0,548
Allgemeine Einschätzung	Bedeutung des Kinderbetreuungsangebots (KidsClub)	Fachrichtung (Kategorien ≥5)	Kruskal-Wallis-Test	220	0,712

Allgemeine Einschätzung	Bedeutung des Kinderbetreuungsangebots (KidsClub)		Institutionstyp (4 Hauptgruppen)	Kruskal-Wallis-Test	220	0,399
Allgemeine Einschätzung	Bewertung von Programmdichte und Parallelveranstaltungen	Zu viele parallele Sessions – ich kann nicht alles verfolgen, was mich interessiert	Berufsstatus (AiW vs. Fach-/Oberärzt:innen)	Fisher-Exact-Test	200	0,183
Allgemeine Einschätzung	Bewertung von Programmdichte und Parallelveranstaltungen	Zu viele parallele Sessions – ich kann nicht alles verfolgen, was mich interessiert	Alter (≤29/30–34/35–40/>40)	Chi-Quadrat-Test	226	0,566
Allgemeine Einschätzung	Bewertung von Programmdichte und Parallelveranstaltungen	Zu viele parallele Sessions – ich kann nicht alles verfolgen, was mich interessiert	Fachrichtung (Kategorien ≥5)	Chi-Quadrat-Test	220	0,126
Allgemeine Einschätzung	Bewertung von Programmdichte und Parallelveranstaltungen	Zu viele parallele Sessions – ich kann nicht alles verfolgen, was mich interessiert	Institutionstyp (4 Hauptgruppen)	Chi-Quadrat-Test	220	0,347
Allgemeine Einschätzung	Bewertung von Programmdichte und Parallelveranstaltungen	Die Anzahl paralleler Sessions ist genau richtig	Berufsstatus (AiW vs. Fach-/Oberärzt:innen)	Fisher-Exact-Test	200	0,859
Allgemeine Einschätzung	Bewertung von Programmdichte und Parallelveranstaltungen	Die Anzahl paralleler Sessions ist genau richtig	Alter (≤29/30–34/35–40/>40)	Chi-Quadrat-Test	226	0,624
Allgemeine Einschätzung	Bewertung von Programmdichte und Parallelveranstaltungen	Die Anzahl paralleler Sessions ist genau richtig	Fachrichtung (Kategorien ≥5)	Chi-Quadrat-Test	220	0,191
Allgemeine Einschätzung	Bewertung von Programmdichte und Parallelveranstaltungen	Die Anzahl paralleler Sessions ist genau richtig	Institutionstyp (4 Hauptgruppen)	Chi-Quadrat-Test	220	0,463
Allgemeine Einschätzung	Bewertung von Programmdichte und Parallelveranstaltungen	Es gibt zu wenig Auswahl / zu wenige Sessions gleichzeitig	Berufsstatus (AiW vs. Fach-/Oberärzt:innen)	Fisher-Exact-Test	200	0,632
Allgemeine Einschätzung	Bewertung von Programmdichte und Parallelveranstaltungen	Es gibt zu wenig Auswahl / zu wenige Sessions gleichzeitig	Alter (≤29/30–34/35–40/>40)	Chi-Quadrat-Test	226	0,098

Allgemeine Einschätzung	Bewertung von Programmdichte und Parallelveranstaltungen	Es gibt zu wenig Auswahl / zu wenige Sessions gleichzeitig	Fachrichtung (Kategorien ≥5)	Chi-Quadrat-Test	220	0,834
Allgemeine Einschätzung	Bewertung von Programmdichte und Parallelveranstaltungen	Es gibt zu wenig Auswahl / zu wenige Sessions gleichzeitig	Institutionstyp (4 Hauptgruppen)	Chi-Quadrat-Test	220	0,487
Allgemeine Einschätzung	Bewertung von Programmdichte und Parallelveranstaltungen	Ich vermisse fachspezifische Angebote in meinem Interessensgebiet	Berufsstatus (AiW vs. Fach-/Oberärzt:innen)	Fisher-Exact-Test	200	0,600
Allgemeine Einschätzung	Bewertung von Programmdichte und Parallelveranstaltungen	Ich vermisse fachspezifische Angebote in meinem Interessensgebiet	Alter (≤29/30–34/35–40/>40)	Chi-Quadrat-Test	226	0,089
Allgemeine Einschätzung	Bewertung von Programmdichte und Parallelveranstaltungen	Ich vermisse fachspezifische Angebote in meinem Interessensgebiet	Fachrichtung (Kategorien ≥5)	Chi-Quadrat-Test	220	0,218
Allgemeine Einschätzung	Bewertung von Programmdichte und Parallelveranstaltungen	Ich vermisse fachspezifische Angebote in meinem Interessensgebiet	Institutionstyp (4 Hauptgruppen)	Chi-Quadrat-Test	220	0,672
Allgemeine Einschätzung	Bewertung von Programmdichte und Parallelveranstaltungen	Ich finde die Balance zwischen allgemeinen und fachspezifischen Themen gut	Berufsstatus (AiW vs. Fach-/Oberärzt:innen)	Fisher-Exact-Test	200	0,735
Allgemeine Einschätzung	Bewertung von Programmdichte und Parallelveranstaltungen	Ich finde die Balance zwischen allgemeinen und fachspezifischen Themen gut	Alter (≤29/30–34/35–40/>40)	Chi-Quadrat-Test	226	1,000
Allgemeine Einschätzung	Bewertung von Programmdichte und Parallelveranstaltungen	Ich finde die Balance zwischen allgemeinen und fachspezifischen Themen gut	Fachrichtung (Kategorien ≥5)	Chi-Quadrat-Test	220	0,992
Allgemeine Einschätzung	Bewertung von Programmdichte und Parallelveranstaltungen	Ich finde die Balance zwischen allgemeinen und fachspezifischen Themen gut	Institutionstyp (4 Hauptgruppen)	Chi-Quadrat-Test	220	0,090
